# Mouse HORMAD1 and HORMAD2, Two Conserved Meiotic Chromosomal Proteins, Are Depleted from Synapsed Chromosome Axes with the Help of TRIP13 AAA-ATPase

**DOI:** 10.1371/journal.pgen.1000702

**Published:** 2009-10-23

**Authors:** Lukasz Wojtasz, Katrin Daniel, Ignasi Roig, Ewelina Bolcun-Filas, Huiling Xu, Verawan Boonsanay, Christian R. Eckmann, Howard J. Cooke, Maria Jasin, Scott Keeney, Michael J. McKay, Attila Toth

**Affiliations:** 1 Institute of Physiological Chemistry, Technische Universität Dresden, Dresden, Germany; 2 Molecular Biology Program, Memorial Sloan-Kettering Cancer Center, New York, New York, United States of America; 3 Cornell University, Ithaca, New York, United States of America; 4 Divisions of Radiation Oncology and Research, Peter MacCallum Cancer Centre, Melbourne, Victoria, Australia; 5 Max Planck Institute of Molecular Cell Biology and Genetics, Dresden, Germany; 6 Medical Research Council Human Genetics Unit, Western General Hospital, Edinburgh, United Kingdom; 7 Developmental Biology Program, Memorial Sloan-Kettering Cancer Center, New York, New York, United States of America; 8 Howard Hughes Medical Institute, New York, New York, United States of America; 9 Department of Radiation Oncology, Australian National University and the Canberra Hospital, Canberra, Australian Capital Territory, Australia; National Cancer Institute, United States of America

## Abstract

Meiotic crossovers are produced when programmed double-strand breaks (DSBs) are repaired by recombination from homologous chromosomes (homologues). In a wide variety of organisms, meiotic HORMA-domain proteins are required to direct DSB repair towards homologues. This inter-homologue bias is required for efficient homology search, homologue alignment, and crossover formation. HORMA-domain proteins are also implicated in other processes related to crossover formation, including DSB formation, inhibition of promiscuous formation of the synaptonemal complex (SC), and the meiotic prophase checkpoint that monitors both DSB processing and SCs. We examined the behavior of two previously uncharacterized meiosis-specific mouse HORMA-domain proteins—HORMAD1 and HORMAD2—in wild-type mice and in mutants defective in DSB processing or SC formation. HORMADs are preferentially associated with unsynapsed chromosome axes throughout meiotic prophase. We observe a strong negative correlation between SC formation and presence of HORMADs on axes, and a positive correlation between the presumptive sites of high checkpoint-kinase ATR activity and hyper-accumulation of HORMADs on axes. HORMADs are not depleted from chromosomes in mutants that lack SCs. In contrast, DSB formation and DSB repair are not absolutely required for depletion of HORMADs from synapsed axes. A simple interpretation of these findings is that SC formation directly or indirectly promotes depletion of HORMADs from chromosome axes. We also find that TRIP13 protein is required for reciprocal distribution of HORMADs and the SYCP1/SC-component along chromosome axes. Similarities in mouse and budding yeast meiosis suggest that TRIP13/Pch2 proteins have a conserved role in establishing mutually exclusive HORMAD-rich and synapsed chromatin domains in both mouse and yeast. Taken together, our observations raise the possibility that involvement of meiotic HORMA-domain proteins in the regulation of homologue interactions is conserved in mammals.

## Author Summary

Generation of haploid gametes in most organisms requires that homologues become connected via crossovers during meiosis. Efficient formation of crossovers depends on HORMA-domain proteins in diverse taxa. These proteins ensure that programmed meiotic DSBs are preferentially repaired from homologues, rather than from sister chromatids. This inter-homologue bias is crucial for homology search and crossovers formation. HORMA-domain proteins have been also implicated in DSB formation, in suppression of synaptonemal complex formation between non-homologous chromosomes, and in the meiotic prophase checkpoint that monitors DSB repair. Despite the importance of HORMA-domain proteins in various organisms, a role for these proteins in mammalian meiosis hasn't been reported. We examined the behaviour of meiotic mouse HORMA-domain proteins—HORMAD1 and HORMAD2—in wild-type and meiotic mutants. HORMAD1/2 preferentially accumulate on unsynapsed chromosome axes. Our data suggest that HORMAD1/2 depletion from chromosomes is a response to synaptonemal complex formation and it that is a conserved process supported by TRIP13/Pch2 AAA-ATPase. Assuming that HORMA-domain functions are conserved in mammals, we speculate that depletion of HORMADs from axes might contribute to the down-regulation of inter-homologue bias and the prophase checkpoint once homology search is completed and synaptonemal complexes form between aligned homologues.

## Introduction

Faithful segregation of chromosomes during the first meiotic division requires that parental homologous kinetochores are physically connected until all pairs of homologous kinetochores attach to microtubules and orient toward opposite spindle poles during metaphase I [1]. Crossovers (COs) in collaboration with sister chromatid cohesion provide these physical connections between maternal and paternal homologues in most organisms, including mammals [1]. Each pair of homologues must have at least one (“obligate”) CO to ensure correct segregation during the first meiotic division.

COs are produced during the first meiotic prophase via recombination. A conserved enzyme, SPO11, introduces double strand breaks (DSBs) into the genome [2]–[4]. DSBs can be repaired using either homologues (inter-homologue repair) or sister chromatids (inter-sister repair) as a recombination/repair template. To ensure CO formation, DSBs are preferentially repaired through the inter-homologue pathway, a phenomenon called the inter-homologue bias (IH bias) [5]. This process also requires that homologous sequences recognise each other. The search for homology is aided by 3′ single-stranded overhangs of resected DSBs, which are produced at the beginning of the repair process [6]. Two RecA homologs, RAD51 and DMC1, assist homology search by promoting strand invasion of resected DNA ends into homologous DNA sequences [7]. The DSB repair process is also coordinated and tightly coupled with dynamic changes in chromatin architecture that facilitate the homology search and stabilise interactions between homologous DNA sequences [1],[8].

One of the key events of meiotic chromosome dynamics is the formation of synaptonemal complexes (SCs) between pre-aligned homologues. SCs play an important role in DSB repair and CO formation [1], [8]–[10]. These proteinaceous structures consist of three parallel elongated elements, two axial elements (AEs) and a central element, which are linked by transverse filaments. The axial element comprises the shared axes of a sister chromatid pair. During SC formation, AEs of homologous chromosomes become connected via the central element/transverse filaments along their entire lengths. AEs begin to form during leptotene prior to the formation of SCs, which starts in zygotene. SCs are fully assembled during pachytene and disassemble as cells progress through diplotene.

SC dynamics and the DSB repair process are coordinated with progression in meiosis. In mammals, spermatocytes with defects in SC formation or DSB repair are eliminated at mid pachytene [11]–[15]. Meiotic silencing of unsynapsed chromosomes (MSUC), in particular the silencing of sex chromosomes, is crucial for normal progression past this arrest point in males [16]–[19]. Due to their restricted homology, X and Y chromosomes are only partially synapsed, and their unsynapsed regions are remodelled into a transcriptionally silenced, phospho-histone H2AX (γH2AX) rich chromatin domain, termed the sex body [16],[20],[21]. Completion of SC formation on autosomes restricts MSUC to sex chromosomes, and is thus essential for full silencing of the X and Y [17],[19]. Spermatocytes with defective SCs exhibit increased expression from sex chromosomes, which is believed to trigger robust elimination by programmed cell death at mid pachytene [19]. In budding yeast, where meiosis is most extensively explored, SC formation and DSB repair are also monitored by a meiotic prophase checkpoint [22],[23].

A conserved feature of the mouse and budding yeast meiotic prophase checkpoints, is the involvement of ATM/ATR-like kinases [22]. In mice, ATR is restricted to unsynapsed chromosome regions during zygotene and pachytene [17]–[19]. Hence, ATR is assumed to phosphorylate H2AX in these regions, resulting in MSUC [17]–[19],[22]. In budding yeast, the ATR and ATM homologs, Mec1 and Tel1, respectively, are required for the prophase checkpoint in collaboration with Hop1, a meiosis-specific HORMA-domain protein (Hop1, Rev7 and MAD2 homology domain) [24],[25].

Hop1 is required for efficient DSB formation, SC formation and the prophase checkpoint [5], [25]–[28]. It also promotes IH bias by inhibiting DSB repair from sister chromatids. [5], [25]–[27],[29]. In particular, phosphorylation of Hop1 by Tel1 and Mec1 is essential for the meiotic prophase checkpoint and for IH bias [25]. Meiotic HORMA-domain proteins are evolutionarily conserved molecules, and they play crucial roles in chromosome behaviour (e.g., SC formation and DSB repair) in other organisms as well, including plants and nematodes [30]–[37]. However, no prior studies have documented involvement of mammalian HORMA-domain proteins in any of the functions known for this important class of proteins.

In an effort to find genes that are specifically involved in meiotic chromosome behaviour in mice, we carried out a screen based on expression profiling of murine meiotic cells. This approach identified *Hormad1* and *Hormad2*, two HORMA-domain encoding genes. HORMAD1 and HORMAD2 proteins are specifically expressed during meiosis in both sexes. We took advantage of characteristic features of mammalian meiosis and powerful cytological methods in mouse in order to better understand the relationships between mouse HORMADs, SC formation, DSB repair, and MSUC.

## Results

### HORMAD1 and HORMAD2 are specifically expressed during meiotic prophase in mice

From expression profiling of reproductive tissues, we found *Hormad1* and *Hormad2* as genes that are up-regulated in female and male gonads when germ cells enter meiosis and progress to the first meiotic prophase (our unpublished results). RT-PCR analysis shows that *Hormad1* and *-2* are not expressed in 17 different somatic tissues, and that both *Hormads* are specifically expressed in the female and male gonads that contain meiotic germ cells (Figure S1A, S1C and S1D). Expression of both genes is restricted to meiotic germ cells in the female gonad at 16.5 days post coitum (dpc) (Figure S1B). Therefore, we conclude that both *Hormad* genes are specifically expressed during meiosis. An earlier study also identified *Hormad1* as a meiosis-specific gene based on *in silico* screening of expressed sequence tags [38].

HORMAD1 and HORMAD2 sequences are similar to the sequences of meiosis-specific HORMA-domain proteins in other organisms (Figure S2). HORMAD1 and -2 are closely related to one another (Figure S2B), and both of them have human homologs (Figure S2A) [39].

To gain insight into the likely meiotic functions of HORMAD1 and -2, antisera were raised against their poorly conserved C-terminal domains (three for HORMAD1 and three for HORMAD2). Antibodies were affinity purified from these antisera (see Materials and Methods). In immunoblot analysis of testis extracts, the anti-HORMAD1 and anti-HORMAD2 antibodies recognised different proteins that have electrophoretic mobilities consistent with the different molecular weights of HORMAD1 and -2, respectively (Figure S3 and Materials and Methods). This suggests that our anti-HORMAD1 and anti-HORMAD2 antibodies recognise HORMAD1 and -2, respectively, and that the antibodies can be used to distinguish between the two HORMADs.

**Figure 1:**
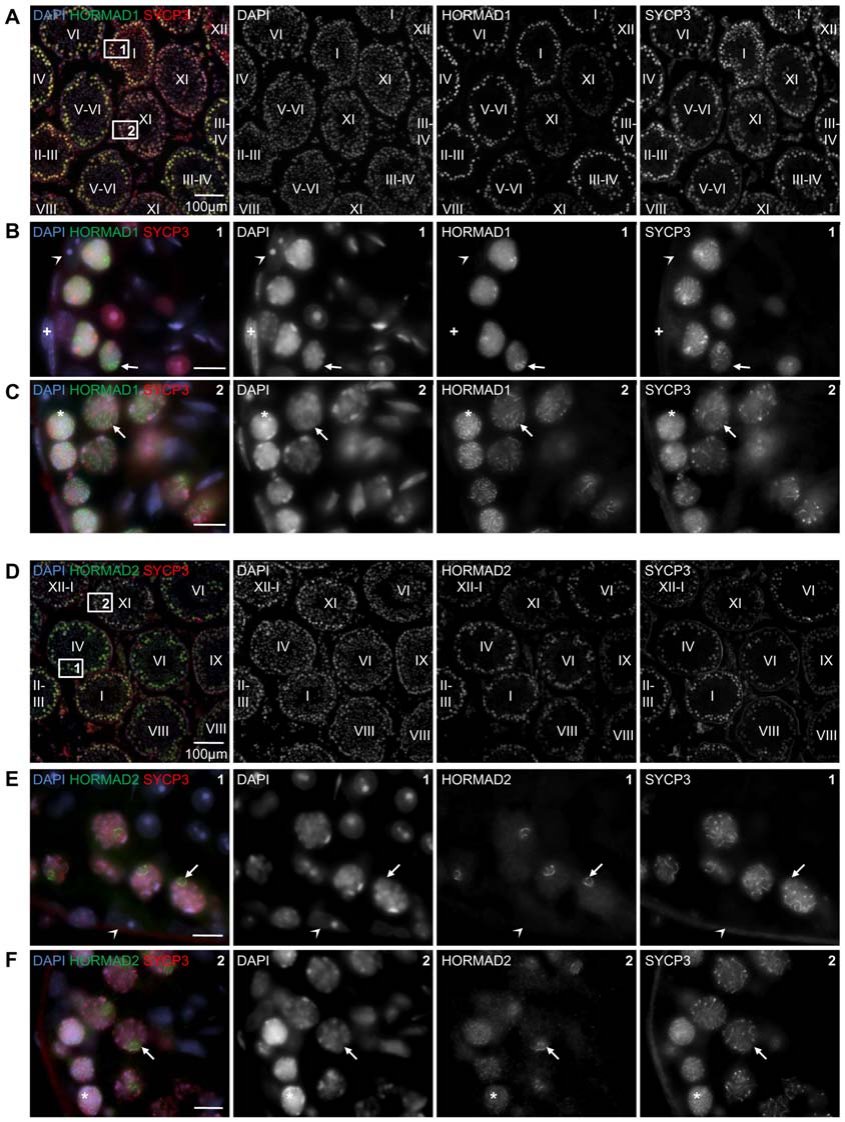
Testicular expression of HORMAD1 and -2 is restricted to meiotic germ cells. Cryo-sections of adult testis were immunostained with anti-SYCP3 and either anti-HORMAD1 (A–C) or anti-HORMAD2 (D–F) antibodies. DNA was detected by DAPI. Overview of several immunostained tubules is shown in A and D. Epithelial cycle stages (Roman numerals) of testis tubules were determined based on SYCP3 localization pattern and DNA staining. HORMAD1/2 levels are highest in nuclei of early/mid pachytene cells (tubule stages I–IV) and decrease as cells progress to late-pachytene and diplotene (tubule stages VIII–XI). Bars are 100 µm in A and D, 10 µm in B, C, E and F. Boxes 1 and 2 from Panel A are shown at higher magnification in B and C. High HORMAD1 levels are detected in the nuclei of spermatocytes marked by chromosome axis component SYCP3. Axis-like staining of HORMAD1 is widespread across nuclei in zygotene (C,*) and diplotene (C, arrow). During pachytene, axis-like staining of HORMAD1 is restricted to a small chromatin domain, which appears to be the sex body based on DNA morphology (B, arrow). Boxes 1 and 2 from Panel D are shown at higher magnification in E and F. HORMAD2 is detected in the nuclei of spermatocytes marked by chromosome axis component SYCP3. Axis-like staining of HORMAD2 is widespread across nuclei in zygotene (F,*). During pachytene (E, arrow) and diplotene (F, arrow) the axis-like staining of HORMAD2 is restricted to a small chromatin domain, which appears to be the sex body. No specific accumulation of HORMAD1 and -2 is observed in sperm, spermatid and any testicular somatic cells, e.g.: Sertoli cells (arrowhead in B and E) and myoid cells (+ in B).

To determine the precise timing of HORMAD1/2 expression during spermatogenesis, we detected HORMADs and the AE component SYCP3 on testis cryo-sections by immunofluorescence (IF) (Figure 1). HORMADs are abundant in the nucleus of spermatocytes throughout the first meiotic prophase, reaching their highest levels during early pachytene. In addition to the general nuclear staining, we observed wide-spread axis-like staining with both anti-HORMAD1 and -2 antibodies during meiotic prophase. No specific staining above background was observed in somatic testicular cells.

### HORMAD1 and HORMAD2 preferentially accumulate on unsynapsed chromosome axes during both female and male meiosis

**Figure 2:**
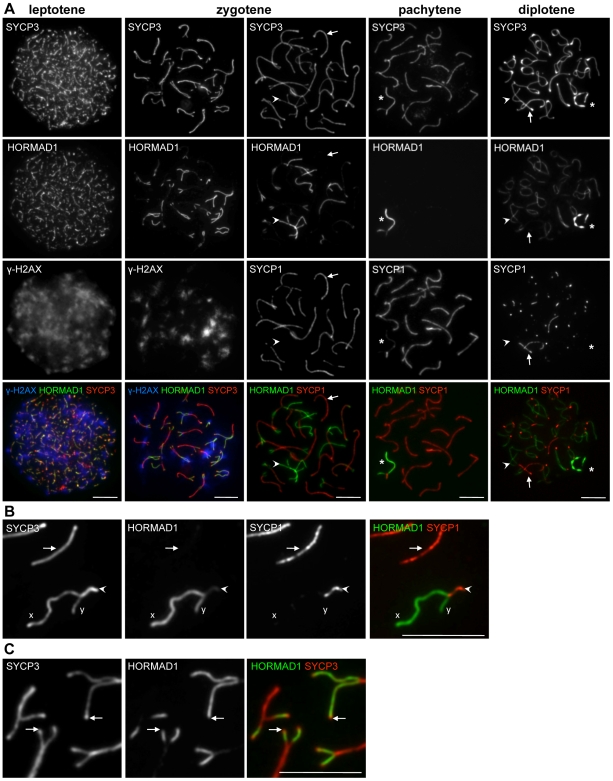
HORMAD1 localization in WT spermatocytes. SYCP3, HORMAD1 and either γH2AX (A, columns 1 and 2) or SYCP1 (A, columns 3–5, B and C) were detected by IF on nuclear surface spreads of WT spermatocytes. Bars, 10 µm. (A) HORMAD1 preferentially associates with unsynapsed chromosome axes (arrowheads). During leptotene and zygotene HORMAD1-enriched chromosome axes overlap with γH2AX-enriched chromatin (columns 1 and 2). Arrows point to synapsed axes in columns 3 and 5. Asterisks mark sex chromosomes in columns 4 and 5. (B) Enlarged view of autosomes and sex chromosomes during pachytene. High levels of HORMAD1 are present on unsynapsed regions of X and Y chromosomes. HORMAD1 signal is reduced more on synapsed autosomes (arrow) than on synapsed regions of sex chromosomes (arrowhead). (C) Enlarged view of zygotene chromosomes. HORMAD1 does not accumulate on SYCP3-rich ends of unsynapsed AEs in the majority of cases (arrows).

To determine the precise dynamics of chromosome association of HORMADs during meiosis, we immunostained surface-spread chromosomes from adult and juvenile (21 dpp) testes for HORMADs and the AE component SYCP3, as well as the transverse filament component SYCP1, γH2AX, RAD51 or centromeres (Figures 2–6). The pattern of SYCP3 staining was used to identify spread spermatocytes at different stages of meiosis.

**Figure 3 pgen-1000702-g003:**
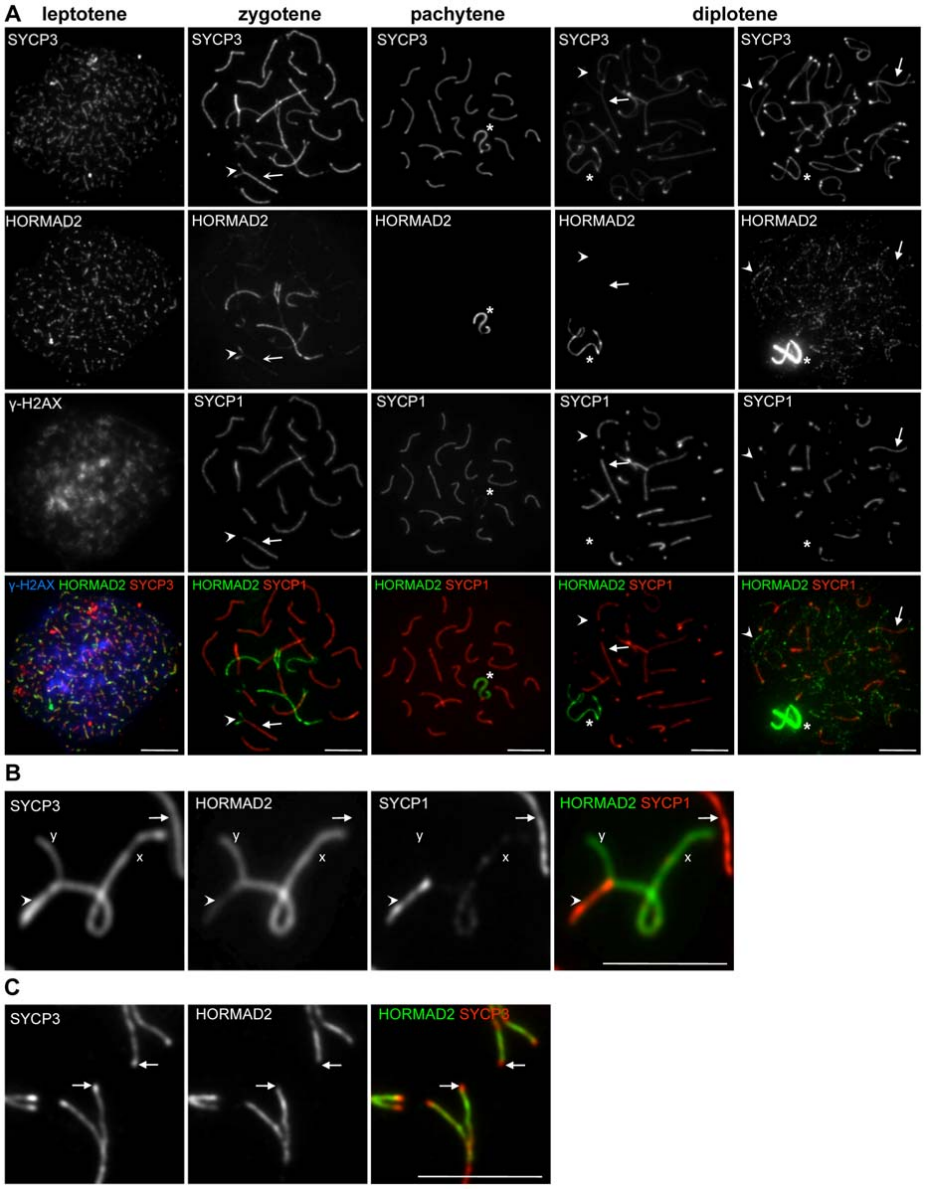
HORMAD2 localization in WT spermatocytes. SYCP3, HORMAD2 and either γH2AX (A, column 1) or SYCP1 (A, columns 2–5, B and C) were detected by IF on nuclear surface spreads of WT spermatocytes. Bars, 10 µm. (A) HORMAD2 preferentially associates with forming AEs and unsynapsed axes during leptotene and zygotene (columns 1 and 2). During pachytene and diplotene, HORMAD2 is present at high levels only on unsynapsed regions of sex chromosomes (asterisks, columns 3 and 4). A slight increase in HORMAD2 staining on desynapsing autosomes can be detected in some diplotene cells on over-exposed images (column 5). Arrows and arrowheads point to synapsed and unsynapsed axes, respectively, in columns 2, 4, and 5. (B) Enlarged view of autosomes and sex chromosomes during pachytene. High levels of HORMAD2 are present on unsynapsed regions of the X and Y. HORMAD2 signal is lower on synapsed autosomes (arrow) than on synapsed regions of sex chromosomes (arrowhead). (C) Enlarged view of zygotene chromosomes. HORMAD2 does not accumulate on SYCP3-rich ends of unsynapsed AEs in the majority of cases (arrows).

**Figure 4 pgen-1000702-g004:**
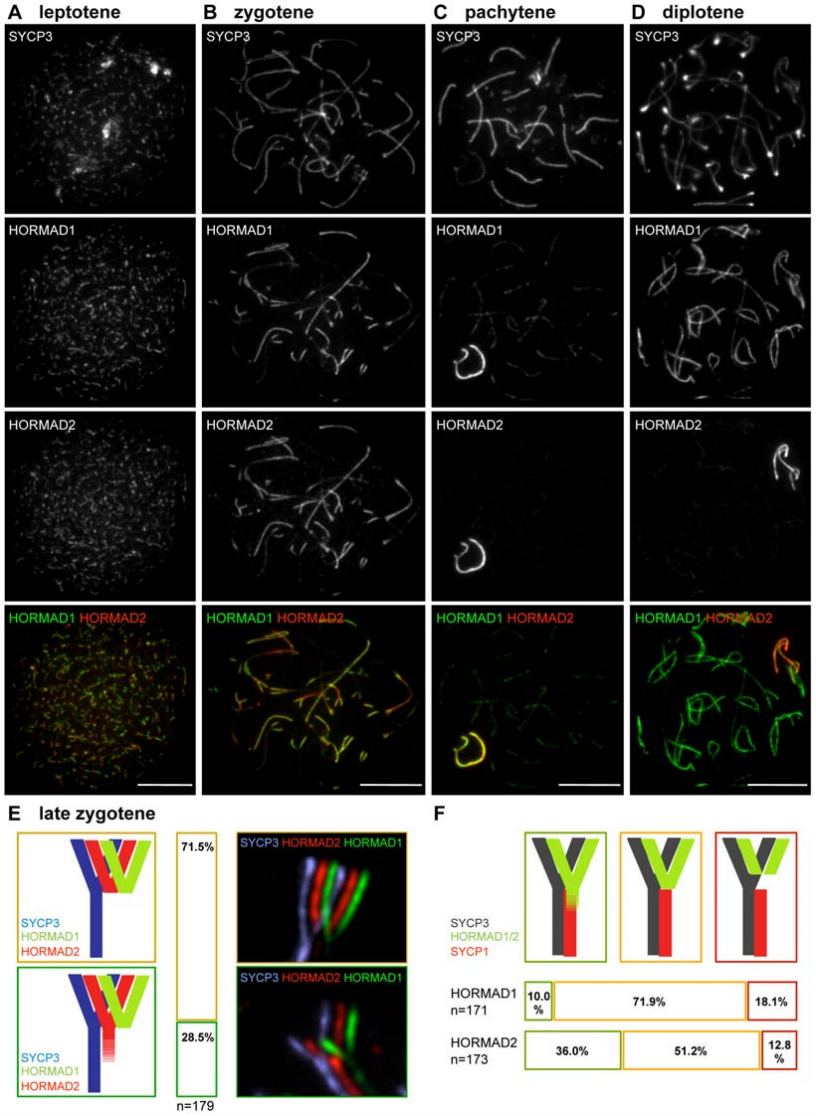
HORMAD1 and HORMAD2 are depleted with different timing from synapsed axes. SYCP3, HORMAD1 and HORMAD2 were detected by IF on nuclear surface spreads of WT spermatocytes of the indicated stages. Both single-channel and merged images are shown. Despite extensive co-localization of HORMAD1 and -2, their patterns differ during zygotene and diplotene. High HORMAD2 levels often persist on synapsed chromosome axes that show little HORMAD1 staining (B). In diplotene, HORMAD1 accumulates to high levels on desynapsing autosomes, while HORMAD2 levels are high only on sex chromosome axes (D). Bars, 10 µm. (E) Quantification of HORMAD1 and -2 localization differences on “Y”-shaped zygotene chromosome axes marked by SYCP3. HORMAD1 and -2 largely co-localize and both appear to be depleted to similar extents from synapsed axes on 71.5% of “Y”-shaped chromosomes (brown-framed cartoon, bar and image). However, in 28.5% of the cases, high HORMAD2 levels are observed further along the synapsed axes (green-framed cartoon, bar and image). (F) Quantification of overlap between high levels of HORMADs on axes and SYCP1 staining. Close to the branching point of ”Y”-shaped zygotene chromosomes, high levels of HORMAD1 and -2 are observed on synapsed axes in 10% and 36% of cases, respectively (green-framed cartoon and bars). HORMAD1 and -2 levels decline at the branching point of ”Y”-shaped zygotene chromosomes in 71.9% and 51.2% of cases, respectively (orange-framed cartoon and bars). HORMAD1 and -2 declines at a distance from the branching point of chromosomes in 18.5% and 12.8% of the cases, respectively (red-framed cartoon and bars).

**Figure 5 pgen-1000702-g005:**
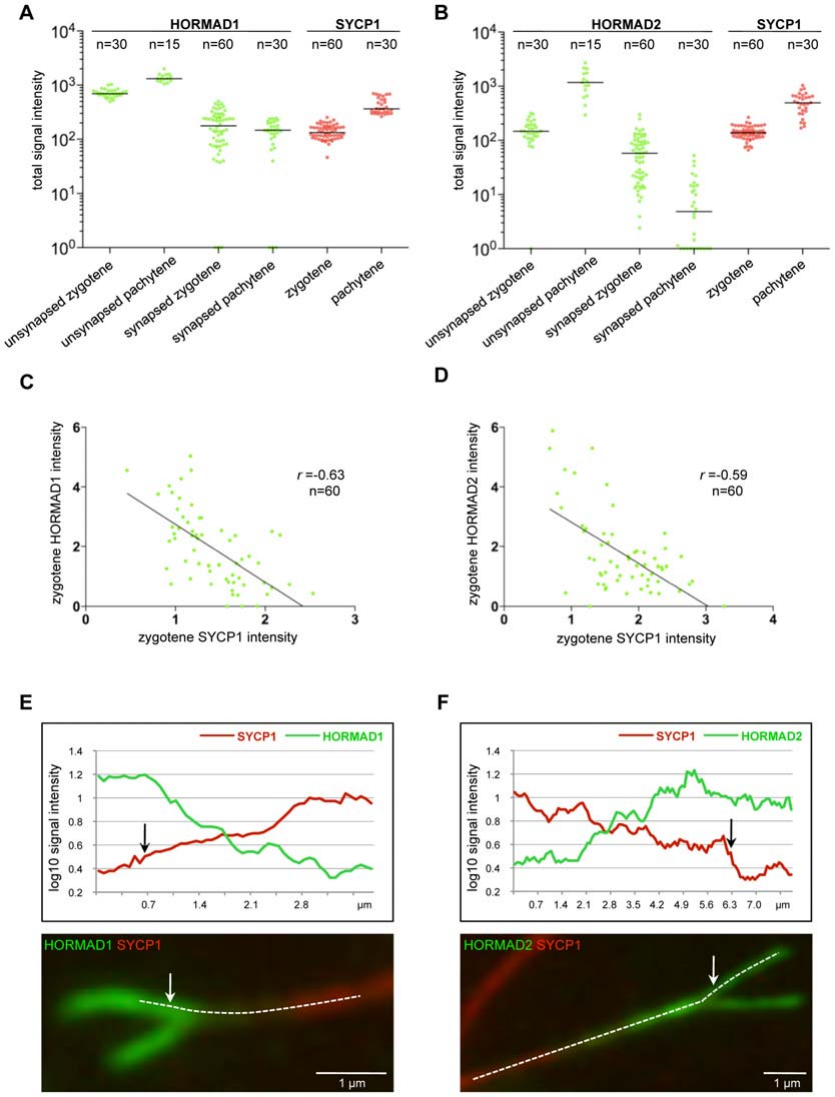
Quantification of axis-associated HORMAD1 and HORMAD2 staining. SYCP3, SYCP1 and either HORMAD1 or -2 were stained on nuclear spreads of WT zygotene and pachytene spermatocytes. Images of 15 cells were captured from each stage with identical camera settings. SYCP1 and HORMAD1 or HORMAD2 IF signal levels were measured on synapsed and unsynapsed chromosome axes (chromosome numbers are indicated on top of the graphs). Background-corrected total signal intensities of HORMAD1 (A, green), HORMAD2 (B, green) and SYCP1 (A and B, red) are shown for each examined chromosome axes at the indicated stages. Units of signal intensities are arbitrary and can not be compared between the two experiments (A and B). Signal intensities are plotted on logarithmic scale to allow comparison of both high and low levels of signal. Chromosomes with background level staining are plotted on the X axis and set to 10^0^ = 1 value. Median signal intensities are marked by horizontal lines. (C, D) Negative correlation between HORMAD1/2 and SYCP1 staining on synapsed axes of zygotene chromosomes. HORMAD1 or HORMAD2 signal intensities are plotted against the SYCP1 signal intensities for synapsed regions. Trendlines, Pearson's *r* values and numbers of chromosomes analyzed are shown in both scatter plots. (E, F) Gradients of HORMAD1/2 staining along synapsing bivalents. Signal intensity profiles of SYCP1 and either HORMAD1 (E) or HORMAD2 (F) along the axes of representative examples of “Y”-shaped chromosomes. The intensity profiles were generated along the dashed lines overlaid on the chromosome images. The chromosome in (F) is an example where HORMAD2 signal intensity is higher on just-synapsed double axes than on unsynapsed single axes. Arrows indicate synaptic branching points.

**Figure 6:**
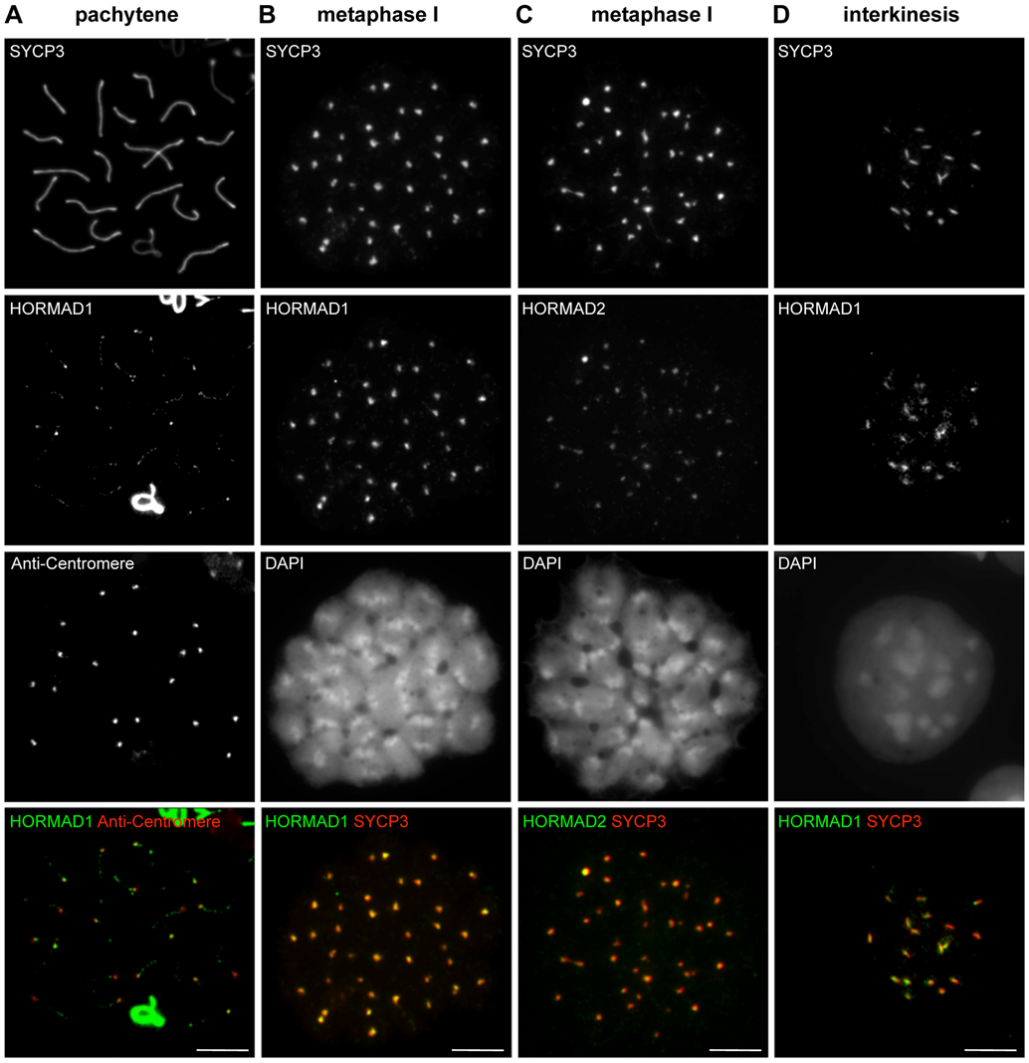
HORMADs associate with centromeres during meiosis. Indicated proteins and centromeres were detected by IF on nuclear spreads of male meiotic cells. In pachytene cells (A), low amounts of HORMAD1 remain on the synapsed autosomes, with the highest signal in the vicinity of centromeres. During the first metaphase (B, C) and interkinesis/second prophase (D), SYCP3 marks centromeres and the region between sister centromeres, respectively. HORMAD1 co-localizes with SYCP3 during both stages (B, D), whereas HORMAD2 is detectable only during metaphase I at SYCP3 foci (C). Bars, 10 µm.

#### Localization of HORMADs in leptotene spermatocytes

HORMADs first appear on chromatin during leptotene, accumulating on short stretches of the forming chromosome axes marked by SYCP3 (Figure 2A and 3A). They extensively co-localize with SYCP3 during leptotene and early zygotene, when homologues start to synapse. Co-staining of HORMAD1 and -2 shows that HORMADs co-localize on chromosome axes although their distribution is not identical (Figure 4A). HORMAD2 IF signals generally appear less intense and more punctate than HORMAD1 signals. RAD51 is believed to associate with newly formed resected DSB ends during leptotene, and it is known to localize to the forming axes [40]. RAD51 foci also co-localize or closely associate with HORMAD1/2 signal on axes during leptotene (Figure S4).

#### Localization of HORMADs in zygotene spermatocytes

Both HORMADs co-localize with SYCP3 on unsynapsed chromosome axes in late zygotene, when SC formation is nearly complete on autosomes (Figures 2A and 3A). Nevertheless, the staining patterns of SYCP3 and HORMADs differ at the ends of AEs, where SYCP3 accumulates to higher levels than along chromosome axes (Figures 2C and 3C; this enrichment is more obvious at the centromeric ends of the acrocentric mouse chromosomes). Neither HORMAD is enriched at these sites (HORMAD1: 99%, n = 411; HORMAD2: 98.3%, n = 247). Indeed, HORMAD1/2 staining usually declines before the end of the SYCP3-marked axes (HORMAD1: 82.3%, n = 364; HORMAD2: 77.9%, n = 258 chromosome ends; Figures 2C and 3C).

During late zygotene, we observed a strong negative correlation between levels of axis-associated HORMAD1/2 staining and SC formation (n>500 cells observed). HORMAD1/2 staining is intense on unsynapsed chromosome axes but is much reduced on synapsed regions marked by SYCP1 (Figures 2A, 3A, 4 and 5). We never observed chromosomes with uniformly high levels of HORMADs along both synapsed and unsynapsed axes in WT cells.

Quantification of the IF signals confirmed that HORMAD1 and -2 levels are significantly lower on synapsed chromosomes than on unsynapsed chromosome axes (Figure 5; Wilcoxon signed-rank test p<0.0001 for both HORMADs). Residual HORMAD1/2 staining on synapsed regions of chromosomes during zygotene is highly variable (Figure 5A). When we compare SYCP1 and HORMAD1/2 staining intensities on synapsed regions of randomly chosen zygotene chromosomes, we find a negative correlation between the residual HORMAD1/2 IF signal and SYCP1 signal (Pearson's *r* ranged from −0.28 to −0.63 for three independent experiments each for HORMAD1 and -2, 60≥n≥30 chromosomes per experiment; Figure 5C, 5D and data not shown) We often observe a decreasing gradient of HORMAD1 and -2 staining along synapsed axes from the branching point of synapsing AEs toward chromosome regions where SCs had assembled earlier (Figures 5E, 5F; HORMAD1: 34.9%, n = 149; HORMAD2: 50.6%, n = 158 chromosomes in 30 and 32 cells, respectively). Interestingly, on a large fraction of zygotene chromosomes with an obvious gradient of HORMADs, we observe an inverse gradient of SYCP1 staining, i.e., SYCP1 is strongest at regions of lowest HORMAD1 and -2 staining (Figures 5E, 5F; 44/53 and 62/80 chromosomes for HORMAD1 and -2, respectively). Thus, maturation of SCs appears to correlate with depletion of HORMADs from chromosome axes.

Co-staining of zygotene nuclear spreads with anti-HORMAD1 and anti-HORMAD2 antibodies reveals a difference in the behaviour of HORMADs at the axis region where homologous AEs enter newly forming SCs on partially synapsed chromosomes (Figure 4B, 4E). HORMAD2 signal is generally higher and spreads further into the synapsed regions than HORMAD1 on a large fraction of chromosomes (Figure 4E). Co-staining of HORMADs and SYCP1 confirms this pattern: we rarely observe HORMAD1 staining at equivalent levels on synapsed and adjacent unsynapsed regions (Figure 4F), whereas HORMAD2 staining is frequently comparable or even higher in synapsed regions compared with adjacent unsynapsed regions (Figure 4F, 5F). These findings suggest that HORMADs are usually depleted from chromosomes after SC formation and that HORMAD1 is depleted faster than HORMAD2.

In a minority of cases, we observe a small gap between the strong HORMAD signal and the SYCP1 signal at the branch point of partially synapsed chromosomes (Figure 4F). This result may indicate that HORMADs can be depleted from axes before SC formation, but it is also possible that this staining pattern is the result of local loss of SC during or prior to chromosome spreading (see below for further discussion).

#### Localization of HORMADs in pachytene spermatocytes

Median HORMAD1/2 signal intensities on synapsed axes decrease slightly as cells progress from zygotene to pachytene (Figures 5A, 5B; Mann-Whitney U test, p = 0.08 (marginally significant) for HORMAD1 and p<0.0096 for HORMAD2), while the intensity of SYCP1 signal increases during this time (Figures 5A, 5B; p<0.0001). Moreover, HORMAD1/2 staining on unsynapsed regions increases in intensity as meiosis progresses from early zygotene to pachytene (Figures 5A, 5B; p<0.0001). Indeed, the strongest HORMAD1/2 staining is on the unsynapsed regions of the sex chromosomes during pachytene and diplotene (Figures 2–5).

Throughout pachytene, residual HORMAD1/2 signal is low but remains detectable as a punctate pattern along synapsed chromosome axes. HORMAD1 signal is also observed at centromeres on synapsed autosomes in most cells (Figure 6A; 95%, n = 100 cells). HORMAD2 signal on synapsed chromosomes is barely detectable and, while it also appears to accumulate slightly at centromeres, the signal is too faint to reliably count the number of cases (data not shown).

The extent of synapsis between X and Y chromosomes changes as cells progress through pachytene [41],[42]. During early pachytene, a large fraction of the Y chromosome axis can synapse (in part non-homologously) with the X chromosome (see Figure 3B for an example), but the length of the synapsed fraction progressively decreases during later stages until only the very ends of the chromosomes appear connected at the end of pachytene. Throughout pachytene, IF signals of both HORMADs are clearly reduced on synapsed regions of sex chromosomes as compared to unsynapsed regions, while SYCP3 remains abundant on synapsed chromosome axes (Figure 2A, 2B, 3A and 3B). Nevertheless, HORMAD1/2 signals are always higher on synapsed regions of sex chromosomes than on synapsed autosomes (n = 100 cells) (Figure 2A, 2B, 3A and 3B).

#### Localization of HORMADs in diplotene spermatocytes

In diplotene, the two HORMADs behave differently (Figures 2A and 3A). HORMAD1 staining greatly increases on desynapsing autosome axes, while strong staining of the sex chromosomes persists (Figure 2A). In contrast, a strong HORMAD2 IF signal is observed only on the X and Y, although we observe a slight increase in the punctuate HORMAD2 staining on desynapsing autosome axes on some diplotene spreads (Figure 3A). Because HORMAD2 signals on autosomes during diplotene are close to background staining levels, the frequency of such cases is influenced by day-to-day variation in the nuclear spreading, and we can not reliably determine if HORMAD2 re-accumulates on desynapsing autosomes in all diplotene cells.

#### Localization of HORMADs in spermatocytes after diplotene

HORMAD1 and -2 are also detected on nuclear spreads prepared from post-diplotene stages (Figure 6). During diakinesis, AEs disassemble and SYCP3 accumulates at inner centromeres [43], eventually appearing as a bar between sister kinetochores during the second meiotic prophase/interkinesis [44]. HORMAD1 parallels the behaviour of SYCP3: it dissociates from chromosome cores and accumulates at centromeres in all spermatocytes during diakinesis (data not shown), and it overlaps almost perfectly with SYCP3 foci at the inner centromeres and in the bar-like structure during metaphase I and interkinesis, respectively (Figures 6B and D). We could detect low levels of HORMAD2 on inner centromeres in all spermatocytes during the first meiotic metaphase/diakinesis (Figure 6C), but not during interkinesis (data not shown).

#### Localization of HORMADs in oocytes

**Figure 7:**
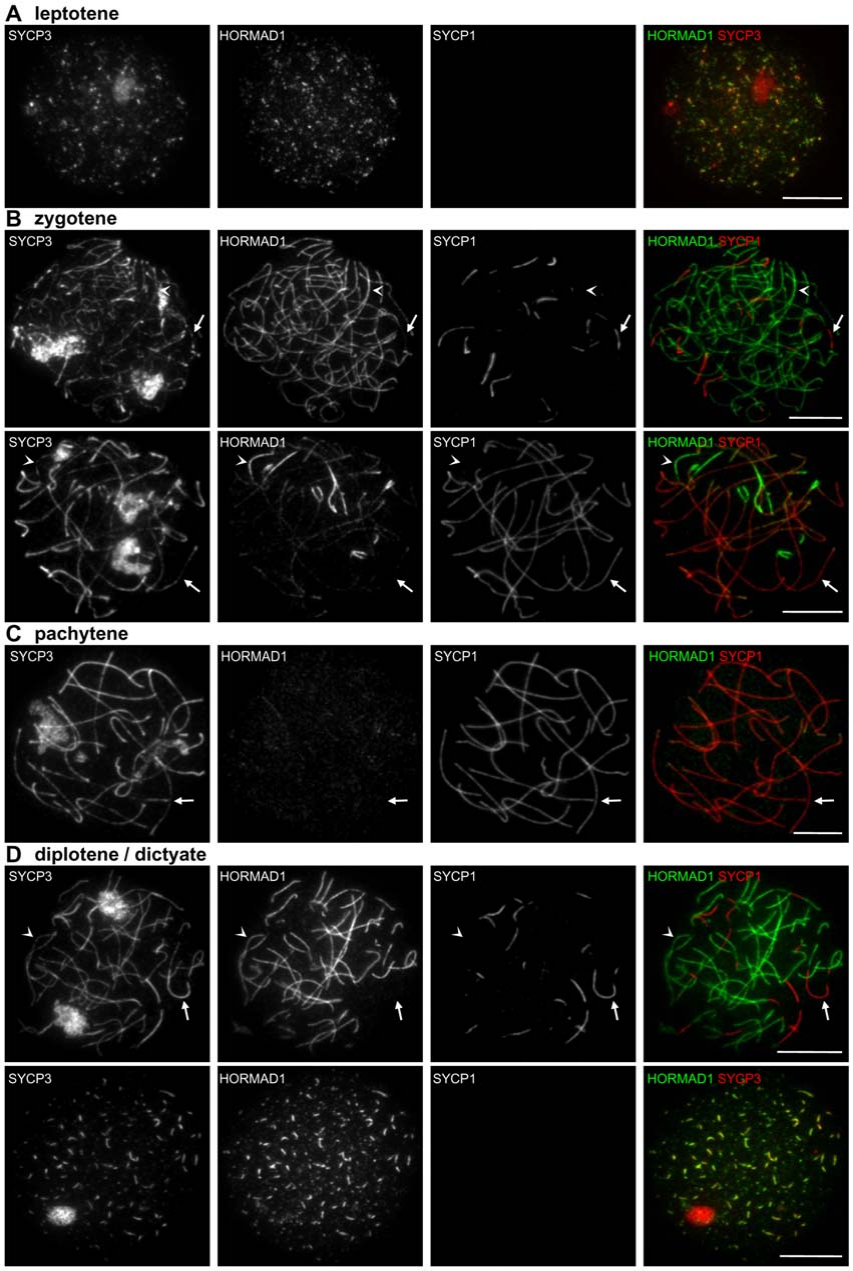
HORMAD1 localization in WT oocytes. SYCP3, HORMAD1 and SYCP1 were detected by IF on nuclear surface spreads of WT oocytes at leptotene (from 16.5 dpc ovaries; A), zygotene (16.5 dpc; B), pachytene (16.5 dpc; C), and diplotene/dictyate (19.5 dpc; D). HORMAD1 preferentially localizes to unsynapsed chromosome axes and is depleted from synapsed axes during zygotene, pachytene and diplotene. HORMAD1 re-accumulates on desynapsing axes in all diplotene stage oocytes (n = 100 cells) and co-localizes with SYCP3 until AEs are disassembled during the dictyate stage (D). Arrows and arrowheads indicate synapsed and unsynapsed axes, respectively. Bars, 10 µm.

**Figure 8:**
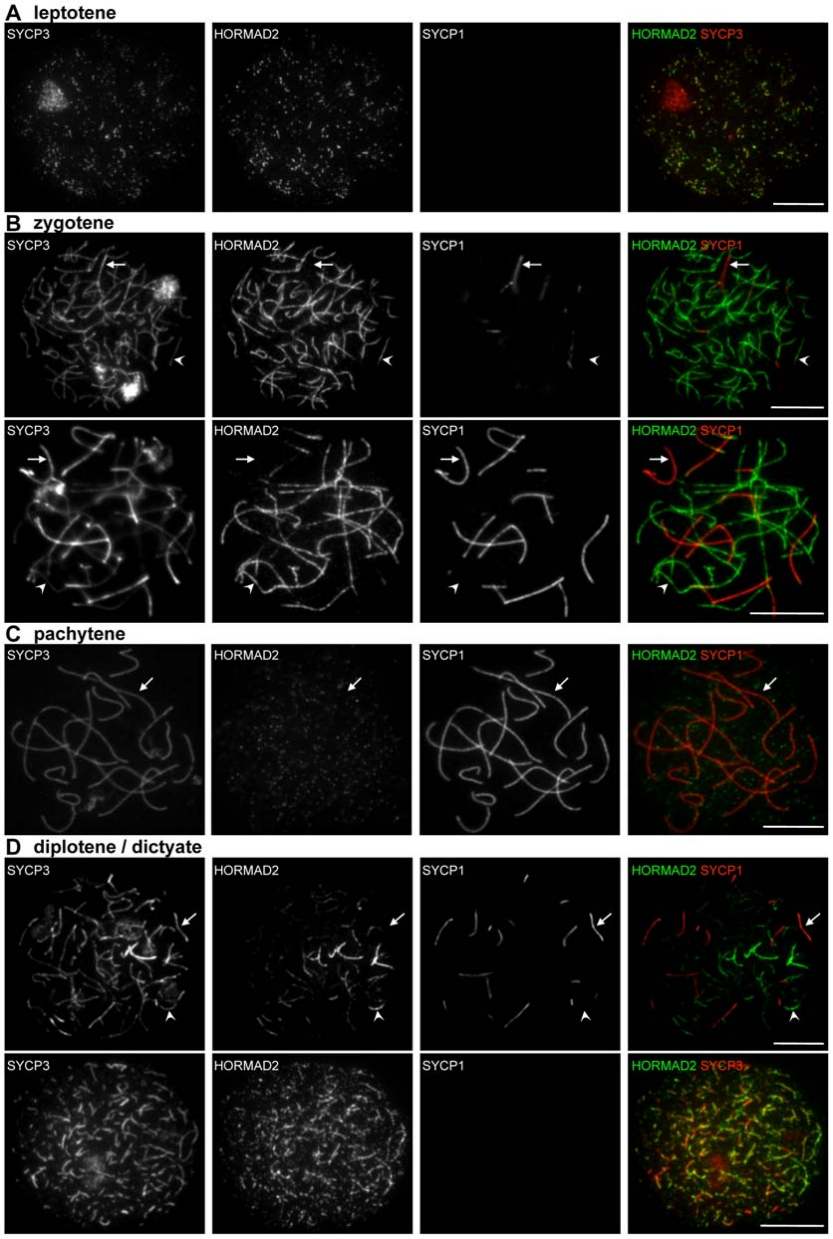
HORMAD2 localization in WT oocytes. SYCP3, HORMAD2 and SYCP1 were detected by IF on nuclear surface spreads of WT oocytes at leptotene (16.5 dpc; A), zygotene (16.5 dpc; B), pachytene (16.5 dpc; C), and diplotene/dictyate (19.5 and 21.5 dpc; D). HORMAD2 preferentially localizes to unsynapsed regions of chromosome axes throughout prophase, and is depleted from synapsed axes during zygotene, pachytene and diplotene. HORMAD2 re-accumulates on desynapsing axes in all diplotene stage oocytes (n = 100 cells) and co-localizes with SYCP3 until AEs are disassembled during the dictyate stage (D). Note that HORMAD2 signal is more uneven and more punctate than HORMAD1 during diplotene (compare with Figure 7D). Arrows and arrowheads indicate synapsed and unsynapsed axes, respectively. Bars, 10 µm.

HORMAD localization in oocytes is similar to patterns in spermatocytes. In females, HORMADs are associated with unsynapsed chromosome axes during leptotene and zygotene (Figures 7, 8). No sex body forms in oocytes because the two X chromosomes fully synapse during pachytene. Accordingly, strong HORMAD staining is not detected on any pachytene chromosomes (Figures 7C, 8C). Unlike in males, substantial levels of both HORMAD1 and HORMAD2 appear on desynapsing axes in all diplotene cells (n = 100 cells) (Figure 7D and 8D). Nevertheless, HORMAD2 staining at this stage is more uneven and less complete than HORMAD1 staining (i.e., some unsynapsed axes do not accumulate high amounts of HORMAD2 (Figure 8D)), and the HORMAD2 staining is also more punctuate than HORMAD1 (compare Figures 7D and 8D). After disassembly of SYCP3-marked AEs in diplotene/dictyate cells, HORMADs cease to accumulate on chromosomes (data not shown).

The similar behaviours and extensive co-localization of HORMAD1 and -2 raise the possibility that the proteins interact. To address this question, we immunoprecipitated HORMAD1 and HORMAD2 from total and nuclear testicular extracts, but we could not detect interaction between HORMADs (data not shown). Nevertheless, we can not exclude the possibility that these two proteins physically interact.

**Figure 9:**
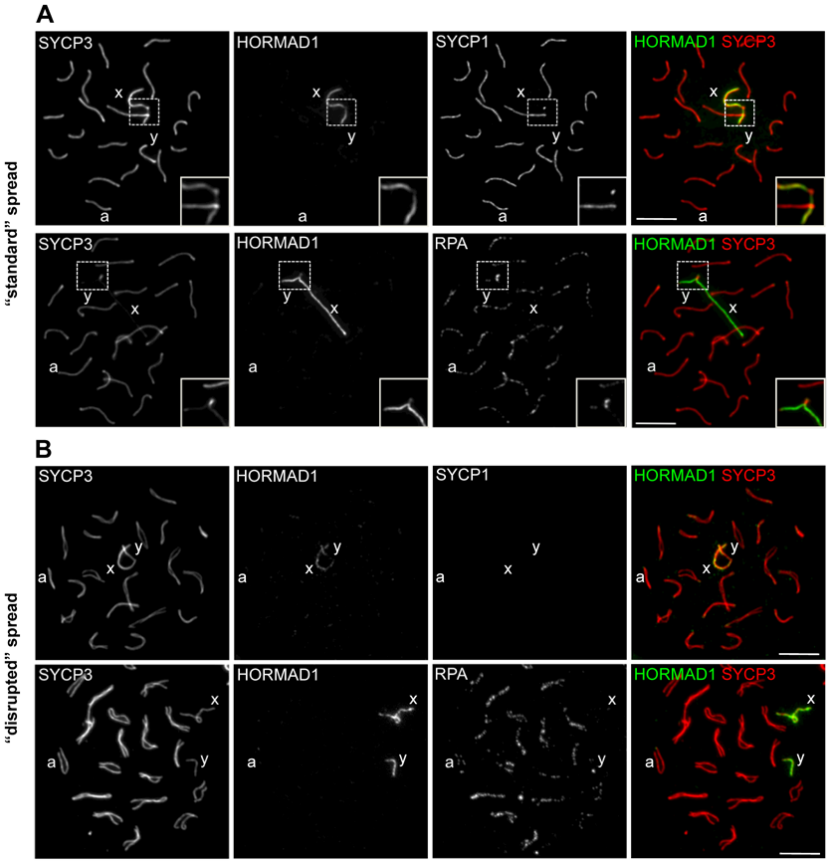
Reduced HORMAD1 staining on synapsed axes is not likely to be a consequence of epitope masking by the SC. “Standard” nuclear surface spreads (A) and “disrupted” nuclear surface spreads (B) were prepared from WT spermatocytes as described in Materials and Methods. Indicated proteins were detected by IF. One autosome and the X and Y chromosomes are marked by a, x and y, respectively. Bars, 10 µm. (A) Immunostaining of “standard” nuclear spreads. Enlarged view of the pseudo-autosomal regions is shown. Anti-HORMAD1 immunostaining is much stronger on unsynapsed sex chromosomes than on synapsed autosomes, which are marked by SYCP1 staining (top row) and by accumulation of RPA (bottom row). Strong RPA accumulation is also observed in the synapsed pseudoautosomal region (enlarged box in bottom row) of sex chromosomes. HORMAD1 level is reduced in this region less than on synapsed autosomes (enlarged box top and bottom row). (B) Immunostaining of “disrupted” nuclear spreads. Autosomes that were synapsed in vivo but that had lost synapsis during spreading were identified either by morphology (upper row) or by high intensity RPA staining (bottom row). Disruption of synapsis due to the modified spreading procedure does not lead to an increase in anti-HORMAD1 staining, with anti-HORMAD1 immunostaining continuing to be much stronger on the sex chromosomes than on the autosomes. Sex chromosomes are identified by either their morphology (top row) or by low levels of RPA (bottom row).

Because strong HORMAD1 staining is observed both before SC formation and after SC disassembly, we considered the possibility that the reduction in HORMAD1 IF signal on synapsed axes is merely a consequence of epitope masking by the SC. To address this issue, we modified our standard spreading protocol and prepared “disrupted” spreads in which SCs break down during sample preparation and autosomal axes that were synapsed within pachytene cells frequently separate from one another on the slides (for more details see Materials and Methods and Figure 9). Desynaptic axes that were synapsed in vivo can be distinguished from unsynapsed autosomes and from the sex chromosomes by staining for RPA, which accumulates preferentially on synapsed axes at DSB sites in zygotene and early/mid pachytene cells [41], [45]–[47] (Figure 9). Importantly, we did not observe HORMAD1 staining on axes where RPA foci were abundant, in either “standard” or “disrupted” nuclear spreads (Figure 9). In addition, we used all three anti-HORMAD1 antibodies for IF staining of 3D-preserved single-cell preparations and nuclear spread preparations of spermatocytes. IF staining of axes was similar irrespective of the antibodies and fixation protocols used (data not shown). These observations, coupled with analysis of the *Trip13* mutant (see below), strongly suggest that the reduction in HORMAD1 staining in synapsed regions is not a trivial consequence of epitope masking by the intact SC.

Taken together, our results indicate that HORMADs are depleted from axes in synapsed chromosome regions during both female and male meiosis, and that both HORMADs re-accumulate on desynapsing axes during diplotene, although re-accumulation of HORMAD2 on autosomes is less obvious in males.

### Depletion of HORMADs from chromosome axes correlates with SC formation in the SC-defective *Smc1β−/−* mutant

**Figure 10:**
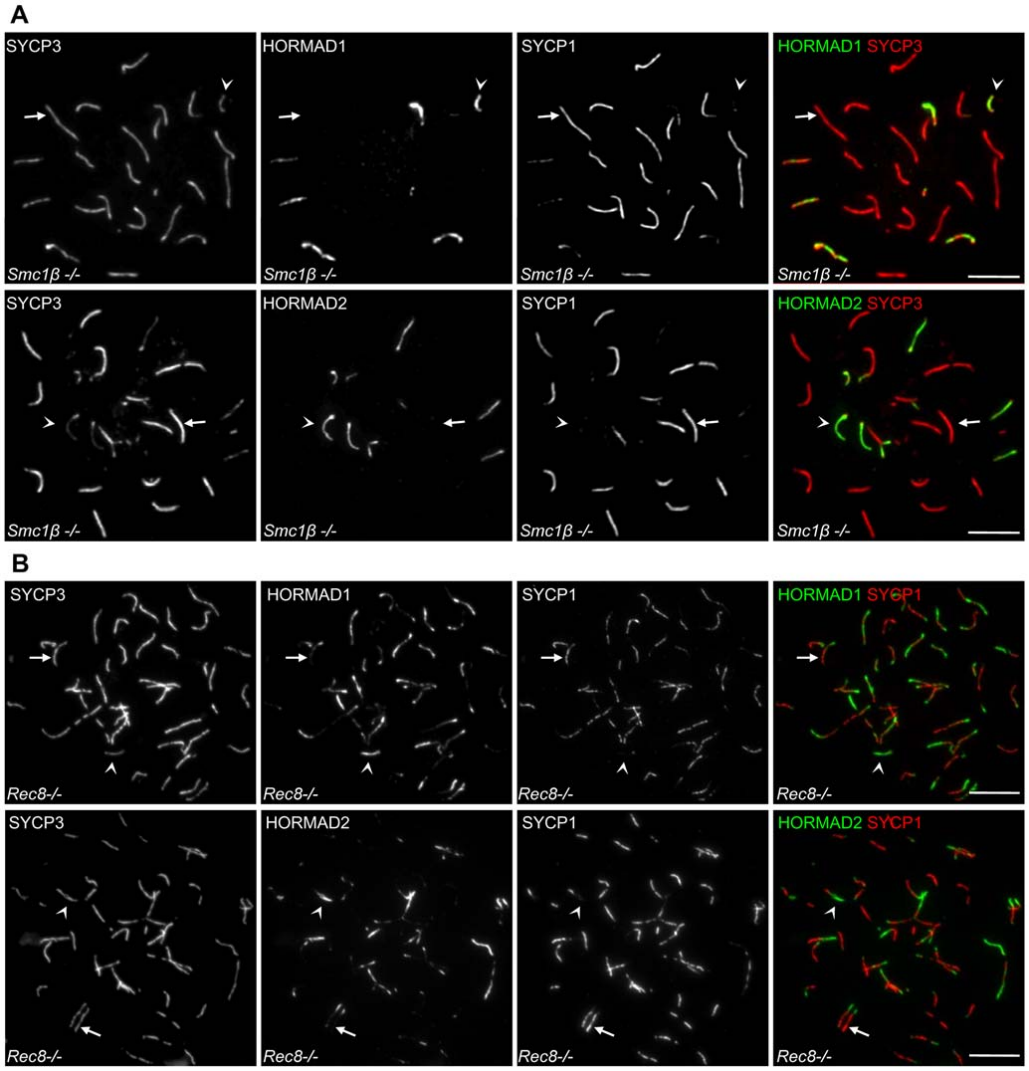
Negative correlation between HORMAD1/2 axis association and SC formation in SC-defective cohesin mutants. (A) IF analysis of nuclear spreads from *Smc1β−/−* spermatocytes. HORMADs persist on unsynapsed axes but are depleted from synapsed regions in cells judged to be equivalent to late zygotene or early pachytene based on the condensation of chromosome axes (n = 100 cells). (B) HORMAD1 and -2 levels are reduced in regions of inappropriate synapsis between sister chromatids in *Rec8−/−* mutant spermatocytes (n = 100 cells). Arrows and arrowheads point to examples of synapsed and unsynapsed chromosome axes, respectively. Bars, 10 µm.

To test if the correlation between HORMAD1/2 depletion and SC formation also exists in cells that fail to form proper SC on all homologue pairs, we examined the behaviour of HORMADs in *Smc1β−/−* spermatocytes, which are partially defective in SC formation [48]. SMC1β is a meiosis-specific mammalian cohesin subunit [49]. In the absence of SMC1β, chromosome axes are shorter than in WT and chromosomes frequently fail to form normal SCs, as evidenced by a lack of SYCP1 along AEs of a subset of condensed chromosomes. Consequently, spermatocytes are eliminated by the mid pachytene checkpoint [48]. In nuclear spreads of *Smc1β −/−* spermatocytes that are at a meiotic stage equivalent to pachytene as judged by their shortened chromosome axes, HORMADs are depleted from synapsed chromosome axes but remain at high levels on abnormally unsynapsed autosomal axes in all mutant “pachytene-like” cells examined (n = 100 cells) (Figure 10A). Thus, we observe a tight correlation between SC formation and local depletion of HORMADs from axes both in WT and in *Smc1β−/−* mutants.

### HORMADs are depleted from chromosome axes that engage in SC formation between illegitimate partners

To further assess the negative correlation between SC formation and axial enrichment of HORMADs, we tested whether HORMAD depletion also occurs in cases where SC forms between illegitimate partners, i.e., between sister chromatids or between non-homologous chromosomes.

REC8 is a meiosis-specific cohesin subunit that is required for normal SC formation between homologues [51],[52]. In the absence of REC8, sister chromatids remain close together during meiotic prophase, suggesting that some form of cohesion still exists [50],[51], but frequently, pairs of sister chromatids form paired AEs, between which SC can form abnormally [51]. Interestingly, we observed high levels of HORMADs only on unsynapsed chromosome axes, whereas HORMADs were depleted from synapsed chromosome axes in all *Rec8−/−* spermatocytes analyzed (n = 100 cells) (Figure 10B). Thus, SC formation between sister chromatids correlates with local depletion of HORMADs from the synapsed chromosome axes.

**Figure 11:**
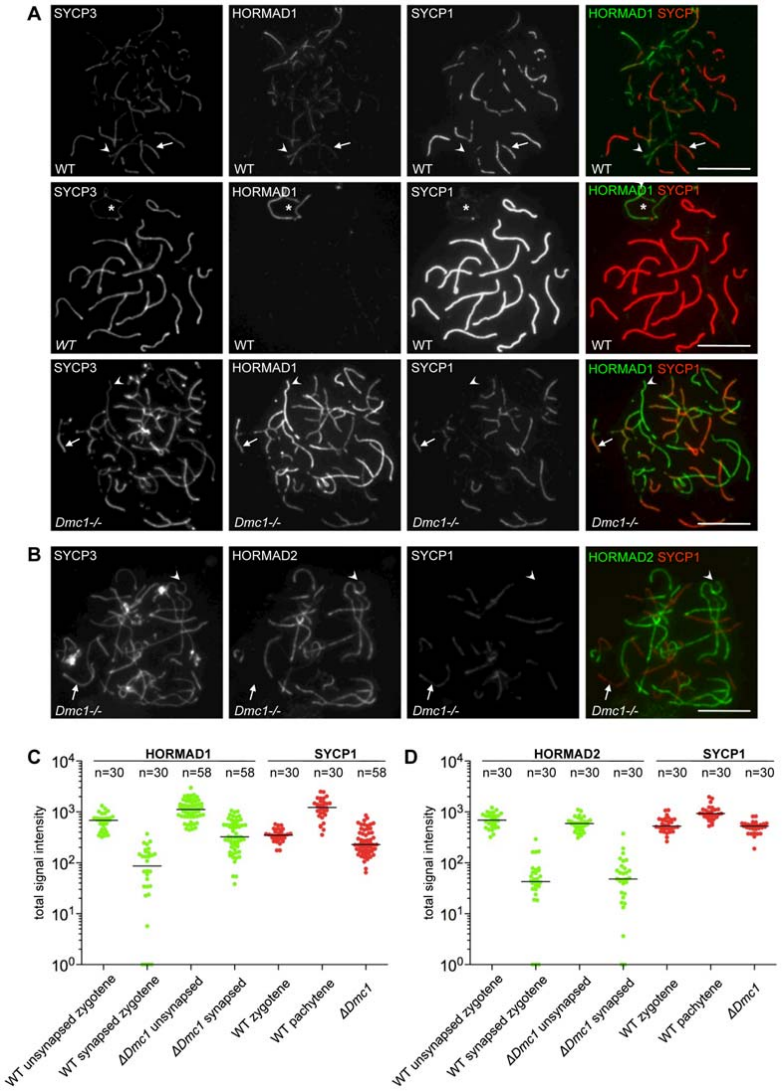
HORMADs are depleted from axes that undergo non-homologous SC formation in *Dmc1−/−* mutants. Indicated proteins were detected by IF on nuclear spreads of WT zygotene (A, top row), WT pachytene (A, middle row) and *Dmc1−/−* (A, bottom row, and panel B) spermatocytes. Arrows and arrowheads indicate examples of synapsed and unsynapsed axes, respectively. Bars, 10 µm. (A) Matched exposures of nuclei that were spread and immunostained in parallel are shown. SYCP1 levels on non-homologously synapsed chromosomes in the mutant are comparable to, or slightly lower than, levels on homologously synapsed chromosomes in WT zygotene cells. Overall, the HORMAD1 signal on both unsynapsed and synapsed axes is higher in *Dmc1−/−* cells than in WT zygotene cells. Nevertheless, HORMAD1 signal is reduced on synapsed axes as compared to unsynapsed axes (n = 100 cells). Asterisk marks sex chromosomes (A, middle row). (B) HORMAD2 signal is depleted from synapsed axes in the mutant (n = 100 cells). (C) and (D) Quantification of SYCP1 and corresponding HORMAD1 or HORMAD2 IF signals on synapsed and unsynapsed chromosome axes (numbers of chromosomes analyzed are indicated on top of the graphs; matched exposures for each category were taken from 15 randomly selected cells for each experiment). Background-corrected total signal intensity of HORMAD1 (C, green), HORMAD2 (D, green) and SYCP1 (C and D, red) are shown for each chromosome axis at the indicated stages. Signal intensity units are arbitrary, and thus can not be compared between panels C and D. Signal intensities are plotted on a logarithmic scale to allow comparison of both high and low levels of signal. Chromosomes with background level staining are plotted on the X axis. Median signal intensities are marked by horizontal lines.

To address if SC formation between non-homologous chromosomes also correlates with depletion of HORMADs, we examined HORMAD1/2 behaviour in *Dmc1−/−* mutant cells (Figure 11). DMC1 is a meiosis-specific RecA homolog. In *Dmc1−/−* mutants, DSBs are produced but can not be repaired efficiently, probably due to a failure in homology search and homologue alignment [52],[53]. Consequently, only short stretches of SCs form, and these tend to connect non-homologous chromosomes [52],[53]. We find that SYCP1 levels on synapsed axes in the *Dmc1−/−* mutant are much lower than in WT pachytene cells (Mann-Whitney U test, p<0.0001; Figures 11A, C, and D). SYCP1 levels in the mutant are comparable to, or slightly lower than, the levels seen in WT cells at zygotene (p = 0.2838 and p = 0.0007 in the HORMAD2 and -1 experiments; Figures 11A, C, and D). These data indicate that *Dmc1−/−* mutant cells do not form fully matured “pachytene”-type SCs. Nevertheless, we found that SC formation in this mutant correlates with a clear reduction in the amount of axis-associated HORMADs, with both HORMAD1 and -2 showing significantly lower signals on synapsed axes than on unsynapsed ones (p<0.0001; Figures 11C, D). Interestingly, staining intensities for HORMAD2 are similar between *Dmc1−/−* mutant and WT zygotene cells for axes with similar synaptic configurations (Figure 11D). In contrast, median HORMAD1 levels are higher in the mutant than in WT zygotene cells (p<0.0001, for both synapsed and unsynapsed axes, Figure 11C). Taken together, these results reveal that DMC1-dependent progression of homologous recombination is not essential for depletion of HORMADs from axes, and we can conclude that both homologous and non-homologous synapsis correlate with local depletion of HORMADs.

### SC central element components are required for depletion of HORMADs from chromosome axes

**Figure 12:**
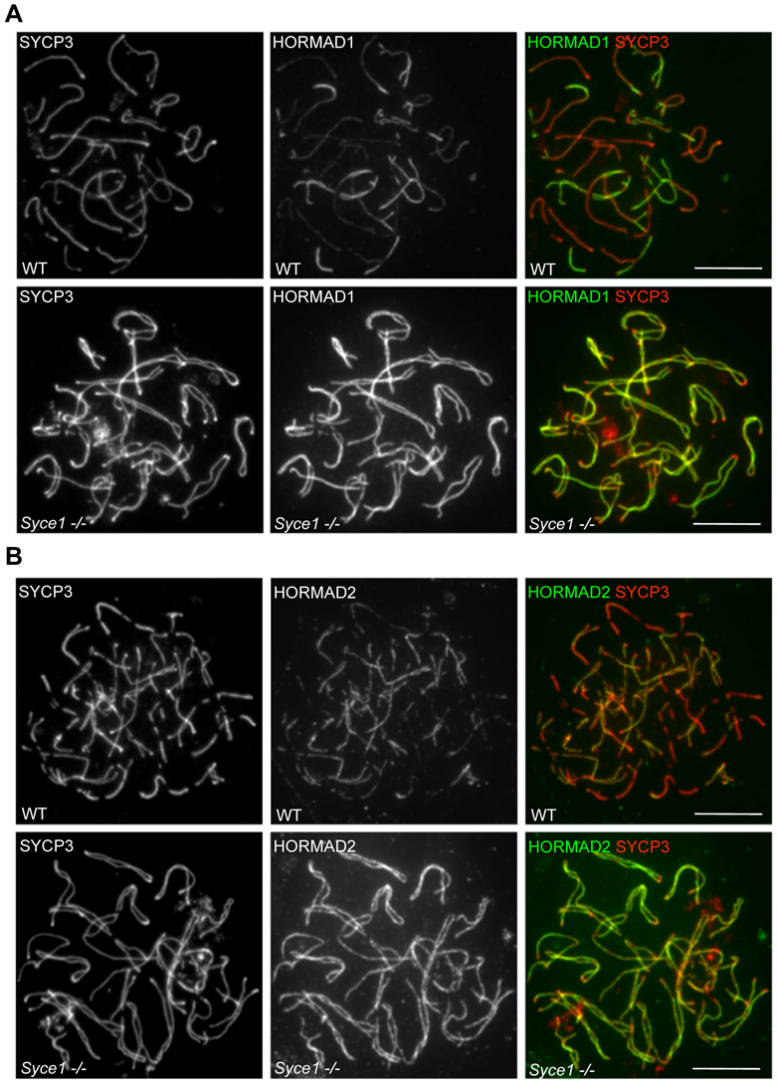
SC formation is required for the depletion of HORMADs from chromosome axes. SYCP3 and either HORMAD1 or HORMAD2 were detected by IF on nuclear spreads of a mixed population of WT and *Syce1−/−* spermatocytes. Matched exposures are shown for WT zygotene (A and B, top rows) and the *Syce1−/−* mutant (A and B, bottom rows). Bars, 10 µm.

To test if SC formation is required for depletion of HORMADs from chromosome axes, we examined mutants lacking SYCE1 and SYCE2, two essential components of the SC central element [11],[14],[54]. Neither *Syce1−/−* nor *Syce2−/−* mutant spermatocytes are able to form stable SCs, and mutant spermatocytes undergo apoptosis at a stage equivalent to mid pachytene [11],[14]. Despite the lack of SC formation, fully formed AEs for each homologue pair appear to align in the mutants [11],[14]. Both HORMAD1 and HORMAD2 are highly enriched on the aligned but unsynapsed axes in all mutant spermatocytes examined (n = 100 cells for each mutant from 6 weeks old animals) (Figure 12, and data not shown for *Syce2−/−*). When chromosome spreads were prepared from mixed populations of WT and mutant cells, direct comparison between cells on the same slides showed that the signal intensity of HORMAD1/2 staining is at least as high on unsynapsed axes of mutant spermatocytes as on unsynapsed axes in WT zygotene cells (n = 100 cells were compared) (Figure 12). In fact, HORMAD1 signal appears to be comparable between unsynapsed axes in the mutant and unsynapsed regions of sex chromosomes in WT during pachytene (data not shown). Thus, there is no depletion of HORMAD1 or HORMAD2 from chromosome axes in mutants lacking SC central element components.

These observations are inconsistent with the idea that SC formation and depletion of HORMADs from synapsed axes are simultaneous, but independently regulated processes in WT. To the contrary, these results suggest that there is a causal relationship between the two events, in which SC formation may directly or indirectly promote local depletion of HORMADs from the axes. Nevertheless, we can not fully exclude the possibility that previously unrecognised SC-independent functions of SYCE1 and SYCE2 are responsible for the depletion of HORMADs concomitant with synapsis.

### DSBs and the protein kinase ATM are not required for axis association of HORMADs

We observed a strong correlation between the accumulation of HORMADs on chromosome axes and the accumulation of γH2AX on neighbouring chromatin in WT cells (Figure 2A and 3A) and in all the previously discussed mutants during pre-diplotene stages (data not shown).

In WT mice, ATM promotes formation of γH2AX in response to SPO11-induced DSBs during leptotene and early zygotene (Figure S5) [15],[55]. ATM is not required during late zygotene and pachytene, when ATR instead is thought to be responsible for formation of γH2AX on unsynapsed chromosomal regions and for meiotic silencing of unsynapsed chromosomes (MSUC) [15],[17],[18],[55]. The correlation between presence of γH2AX and the initial accumulation of HORMADs on axes may reflect a causal relationship between HORMAD localization and DSB formation and/or ATM/ATR activity. To test this possibility, we examined *Spo11−/−* and *Atm−/−* mutant spermatocytes [2],[3],[56] (Figure S5, 13). In *Spo11−/−* mutants, DSBs do not form and, as a consequence, ATM does not phosphorylate H2AX during early meiotic prophase [15],[20],[55]. HORMADs associate normally with the developing chromosome axes in leptotene and early zygotene spermatocytes in both *Spo11−/−* and *Atm−/−* mutants (Figure S5). Hence, neither DSBs nor ATM activity is required for accumulation of HORMADs on chromosome axes in early prophase.

### DSB formation and repair are not required for depletion of HORMADs from synapsed axes

**Figure 13:**
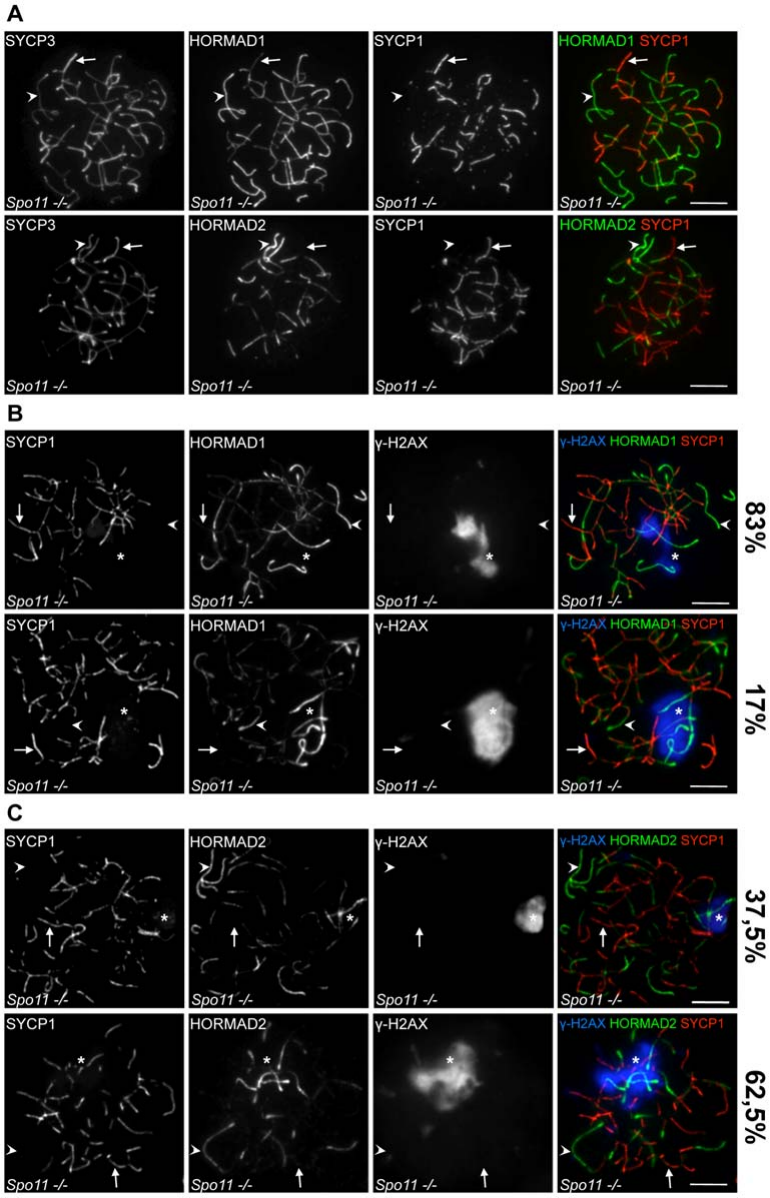
HORMAD localization patterns in cells lacking SPO11-dependent DSB formation. The indicated proteins (SYCP3, SYCP1, γH2AX, and HORMAD1 and -2) were detected by IF on nuclear spreads of *Spo11−/−* spermatocytes. Examples are indicated of synapsed axes (arrows) and unsynapsed axes (arrowheads). Asterisks indicate pseudo-sex bodies. Bars, 10 µm. (A) Levels of both HORMADs are reduced in regions where SC formation has occurred, even though the SC is frequently if not exclusively between non-homologous axes (n = 100 cells examined). (B, C) HORMAD1/2 levels are higher on unsynapsed vs. synapsed axes both within and outside pseudo-sex bodies, but show hyper-accumulation on axes within pseudo-sex bodies in a subset of cells. The top rows of panels B and C show examples of “pachytene-like” cells (i.e., cells with a pseudo-sex body and extensive synapsis) that have comparable HORMAD levels within vs. outside of the pseudo-sex body. The bottom rows show examples of “pachytene-like” cells that have elevated HORMAD staining of axes within the pseudo-sex body. The percentage of cells in each category is indicated (n = 199 for HORMAD1, n = 144 for HORMAD2).

Despite extensive asynapsis and elimination of spermatocytes by the mid pachytene checkpoint, incomplete SCs can form in the *Spo11−/−* mutant [2],[3],[15]. In the absence of DSBs, homologues do not pair and align with one another and SCs frequently form between non-homologous chromosome axes [2],[3]. Similar to *Dmc1−/−* mutants, the intensity of SYCP1 staining in *Spo11−/−* cells is comparable to staining in WT zygotene cells, and is much lower than in WT pachytene cells, indicating that mature “pachytene-type” SCs do not form in *Spo11−/−* mutants (Figure S6). Nevertheless, combined immunostaining of HORMADs, SYCP3 and SYCP1 shows that both HORMADs are depleted from synapsed chromosome axes in mutant cells (n = 100 cells) (Figure 13A and S6). Similar to *Dmc1−/−* mutants, HORMAD1 levels on both synapsed and unsynapsed chromosomes appear higher in the *Spo11−/−* mutant than in WT zygotene cells (Figure S6). We conclude that neither DSB formation nor ongoing recombination is essential for reciprocal distribution of SYCP1 and HORMADs along chromosome axes.

### HORMAD association with unsynapsed axes does not require ATR activity or MSUC

ATR, TOPBP1 (an activator of ATR) and γH2AX frequently accumulate in a restricted chromatin domain in *Spo11−/−* spermatocytes at a stage that is believed to be equivalent to pachytene (i.e., in which a significant amount of SC formation is evident) [15],[19],[20],[55]. This γH2AX-rich domain was termed the pseudo-sex body because it resembles the transcriptionally silenced sex body in WT spermatocytes but rarely overlaps with the sex chromosomes [15],[19],[55]. The pseudo-sex body overlaps with a subset of unsynapsed chromosome axes and effective MSUC is observed in these regions [19]. It is believed that spatially restricted ATR activity is responsible for the formation of pseudo-sex body [15],[19],[55]. Importantly, many unsynapsed chromosome axes do not overlap with the pseudo-sex body in *Spo11−/−* spermatocytes [19]. This situation permitted us to test if ATR activity, marked by accumulation of γH2AX, is needed for preferential accumulation of HORMADs on unsynapsed chromosomes.

Combined IF of HORMADs, SYCP1 and γH2AX in *Spo11−/−* spermatocytes shows that levels of HORMADs are higher on unsynapsed axes than on synapsed axes both inside and outside of pseudo-sex bodies in all observed cells (Figure 13B, 13C). This result suggests that ATR activity and MSUC are not a prerequisite for HORMAD1/2 accumulation on unsynapsed chromosomes. Such a conclusion is also consistent with the published localization pattern of ATR and TOPBP1 in WT spermatocytes. These two proteins co-localize during meiosis [57]. During zygotene, they appear as dot-like foci on unsynapsed axes, whereas during pachytene, they continuously coat the unsynapsed sex chromosome axes and spread to the surrounding silenced sex chromatin [18],[45],[57]. Overall, this localization patter is similar to that of HORMADs in that all of these proteins are restricted to unsynapsed AEs during zygotene and pachytene [18],[57]. Importantly, however, HORMAD1/2 staining coats unsynapsed axes more continuously than TOPBP1 during zygotene (Figure S7A). This difference further supports the idea that axis association of HORMADs is not dependent on ATR activity and MSUC.

### Hyper-accumulation of HORMADs on axes correlates with high levels of ATR activity and MSUC

Although HORMAD levels are always higher on unsynapsed than synapsed axes in *Spo11−/−* cells that formed full-length AEs, HORMADs accumulate to especially high levels on a localized subset of unsynapsed axes within a significant fraction of the mutant cells (bottom rows of Figures 13B and 13C). This phenomenon is more frequently observed for HORMAD2. Importantly, the chromosome axes that have very high HORMAD staining are nearly always located within the pseudo-sex bodies of such cells (99/102 cells for HORMAD1 and 41/42 cells for HORMAD2) (Figures 13B and 13C).

When we examined mutant cells with pseudo-sex bodies more closely, we found three predominant patterns of HORMAD1/2 staining relating to SC formation and developmental stage. In a minority of cells, little SC formation is observed (34%, n = 397), suggesting that these cells are equivalent to late zygotene/early pachytene in WT [15]. In these cells, all unsynapsed axes display comparable HORMAD levels (n = 72 and n = 64 for HORMAD1 and -2, respectively) (data not shown). The remainder of cells have more extensive (non-homologous) SC formation, from which we infer that these cells represent a more advanced developmental stage. These cells can be subdivided into two groups, depending on whether HORMAD levels on unsynapsed axes are comparable inside vs. outside pseudo-sex bodies (top rows in Figures 13B and 13C), or HORMAD levels are substantially elevated within pseudo-sex bodies as compared to axes that lie outside (bottom rows in Figure 13B and 13C). A similar pattern is observed if pseudo-sex bodies are detected by accumulation of TOPBP1 instead of γH2AX (Figures S7B and S7C).

Because pseudo-sex bodies are readily detected in cells without hyper-accumulation of HORMADs, we can conclude that pseudo-sex body formation and the restriction of ATR activity to a subset of unsynapsed chromosomes in *Spo11−/−* mutants is unlikely to be a downstream consequence of additional accumulation of HORMADs. The fact that HORMAD hyper-accumulation (when it is seen) is nearly always associated with pseudo-sex bodies raises the possibility that the presence of high ATR activity and/or other MSUC components supports the accumulation of increased amounts of HORMADs on unsynapsed chromosome axes at more advanced stages of meiosis. We speculate that this pattern in *Spo11−/−* cells might be related to the hyper-accumulation of HORMADs on unsynapsed portions of the X and Y within the sex body in normal cells (e.g., Figures 2 and 3).

### TRIP13 is necessary for the depletion of HORMADs from synapsed regions

**Figure 14:**
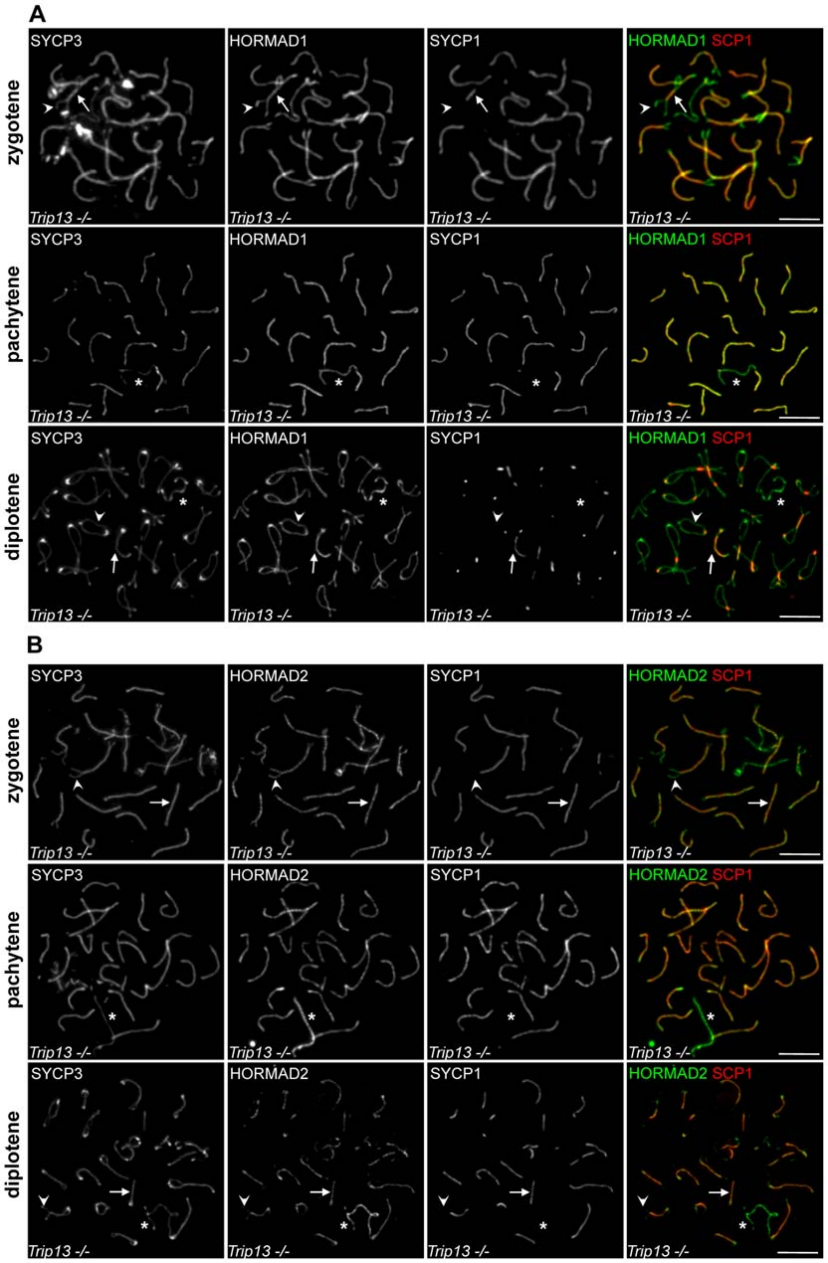
TRIP13 is required for depletion of HORMADs from synapsed chromosome axes. SYCP3, SYCP1 and either HORMAD1 (A) or HORMAD2 (B) were detected on nuclear spreads of *Trip13^hypo^* spermatocytes by IF. High levels of HORMADs remain associated with chromosome axes following SC formation during zygotene and pachytene. No additional accumulation of HORMAD1 and -2 can be observed on desynapsing chromosome axes during diplotene. Arrows and arrowheads point to examples of synapsed and unsynapsed axes, respectively. Asterisks mark the sex chromosomes. Bars, 10 µm.

Localization of budding yeast Hop1/HORMA-domain protein is regulated by the pachytene checkpoint 2 protein (Pch2) [58],[59]. Specifically, Pch2 is required for the depletion of Hop1 from chromosomal regions where Zip1, the yeast transverse filament protein, is abundant [58]. Pch2 is also required for one branch of the meiotic prophase checkpoint and for timely repair of DSBs [58]–[60]. The mouse Pch2 homolog is TRIP13. Analysis of animals homozygous for a hypomorphic mutation (*Trip13^RRB047/RRB047^*; abbreviated *Trip13^hypo^* for simplicity) indicates that TRIP13 is required for timely and efficient repair of meiotic DSBs, but appears to have no meiotic checkpoint function [61] (unpublished data of I. Roig, M. Jasin and S. Keeney). In spite of defective DSB repair, *Trip13^hypo^* spermatocytes form apparently normal looking SCs [61]. To test if PCH2/TRIP13 has a conserved role in the regulation of HORMA-domain proteins, we examined HORMAD localization in the *Trip13^hypo^* mutant. Remarkably, both HORMADs remain detectable at high levels on synapsed axes in all examined mutant spermatocytes during zygotene (n = 100) and in the vast majority of mutant spermatocytes during pachytene (HORMAD1 100%, n = 300; HORMAD2 99,2%, n = 500 cells) (Figure 14). HORMADs persist on desynapsing axes during diplotene but do not accumulate further. The persistently high level of HORMAD1/2 staining in areas where SC has formed in this mutant is unlike any of the patterns observed in WT or any of the other mutants examined. Therefore, we conclude that TRIP13 is required for the normal depletion of HORMADs from synapsed chromosome axes. Furthermore, the ability to readily detect HORMADs in synapsed regions in the *Trip13^hypo^* mutant further reinforces the conclusion that the normal pattern of HORMAD depletion observed in WT and many mutants is not a trivial consequence of epitope masking by the SC (see above).

Interestingly, although there is a clear defect in depletion of HORMAD2 from synapsed axes in the *Trip13^hypo^* mutant, HORMAD2 levels nevertheless do appear higher on the unsynapsed sex chromosomes than on synapsed autosomes in cells where autosomal SC formation is complete (Figure 14B, second row). Similar enrichment on the sex chromosomes is not apparent for HORMAD1 (Figure 14A, second row). Most *Trip13^hypo^* spermatocytes undergo apoptosis during pachytene, but because the mutation reduces but does not fully eliminate *Trip13* gene expression, a small subset of cells are able to progress further, to diplotene and beyond ([61], and unpublished data of I.R., M.J., and S.K.). Hence, it is possible that the slight enrichment of HORMAD2 on sex chromosomes is a consequence of residual TRIP13 activity in a subset of *Trip13^hypo^* spermatocytes that are able to advance the furthest in meiosis. Alternatively, it may be that restriction of high ATR activity to the sex chromosomes during sex body formation, which occurs apparently normally in the majority of pachytene *Trip13^hypo^* spermatocytes, promotes additional accumulation of HORMAD2 on sex chromosome axes in spite of a general defect in depletion of HORMAD2 from synapsed regions. The latter interpretation is consistent with the frequent hyper-accumulation of HORMAD2 observed on unsynapsed axes within pseudo-sex bodies of *Spo11−/−* spermatocytes (see above).

## Discussion

From studies in yeasts, plants, and nematodes, meiosis-specific HORMA domain proteins have emerged as conserved factors that play critical roles in many aspects of the chromosome dynamics important for accurate chromosome segregation [25]–[37]. However, mammalian orthologs of this centrally important class of proteins had not yet been characterised. We address this lack in this study, taking advantage of the powerful cytological tools available for analysis of protein localization to chromosomes and chromosomal arrangements during early stages of meiosis in mammalian germ cells. Our analysis of two mouse HORMA-domain proteins, HORMAD1 and HORMAD2, allowed us to dissect relationships between their behaviour, SC formation, DSB repair and ATR activity/MSUC.

### Relationship between HORMADs and the SC

One of the most striking findings from this study is the pronounced depletion of HORMADs from chromosome axes that have undergone SC formation, both in WT and in mutants where SCs form inefficiently and/or form between illegitimate partners (non-homologous chromosomes or sister chromatids). Several mechanisms, not mutually exclusive, could underlie this inverse correlation between HORMAD localization and the SC. First, SC formation and HORMAD1/2 depletion could be promoted concurrently and independently by another process, such as progression through meiosis, homologue alignment, and/or early DSB repair steps. Alternatively, there may be a causal relationship between SC formation and localized HORMAD1/2 depletion. In this case, either SC formation promotes HORMAD1/2 depletion (directly or indirectly), or axis-associated HORMADs antagonize SC formation.

In *C. elegans*, the HORMA-domain protein HTP-1 and the SC component SYP-1 acquire an almost mutually exclusive, reciprocal localization pattern at the end of pachytene [37], and HTP-1 is also required to prevent premature SC formation between non-homologous chromosomes, which suggests that HTP-1 may inhibit SC formation under some conditions [32],[33]. A similar relationship might exist between SCs and axis-associated HORMADs in mice, but such putative inhibition of SC formation does not provide a straightforward explanation for HORMAD1/2 behaviour in the mutants examined in this study. For example, HORMADs are not depleted from axes in the absence of SC central element components SYCE1 and -2 (Figure 12). If HORMAD depletion is a necessary upstream precondition for SC formation, it is not obvious why HORMAD depletion would be blocked by the absence of central element components, which presumably would cause a relatively late block in SC formation. Moreover, the robust formation of SC in the *Trip13^hypo^* mutant [61], despite persistent axial HORMAD localization (Figure 14), argues against HORMAD depletion being a strict prerequisite for synapsis.

The *Syce1* and *Syce2* mutant phenotypes also argue against the possibility that HORMAD depletion and SC formation are independently induced by another process. For example, extensive AE development, pairing and alignment of homologues, and early steps in DSB repair upstream of SC formation all appear to occur in a timely fashion in these mutants [11],[14], implying that these events are not sufficient to trigger HORMAD depletion. Moreover, it seems unlikely that the spermatogenic arrest in *Syce1* and *Syce2* mutants can account for the defect in HORMAD depletion, because *Dmc1*, *Spo11*, *Smc1*, and *Rec8* mutants also undergo spermatogenic arrest [13],[15],[48],[50],[51], yet these mutants successfully achieve HORMAD depletion in regions where SC has formed.

In summary, although we cannot exclude alternative explanations for the inverse correlation between SC formation and HORMAD localization, we favour the interpretation that SC formation itself, directly or indirectly, promotes localized depletion of HORMADs from synapsed axes.

### Role of SC formation and DSB repair in depletion of HORMADs from AEs

If SC formation does promote depletion of HORMADs from AEs, this could occur through various mechanisms. One possibility could be that SC formation is needed indirectly for meiotic “cell cycle” progression to a zygotene/pachytene-like stage that is permissive for HORMAD1/2 depletion. This possibility seems unlikely, however. WT cells complete full-length AE assembly only in late zygotene, after SCs have already formed along a large fraction of chromosomes. In *Syce1* and *Syce2* mutants, full-length AEs assemble and align in a large fraction of spermatocytes, which indicates that these cells reach a stage that is equivalent to late zygotene/early pachytene [11],[14]. Since WT cells at this stage would have commenced depletion of HORMADs, we infer that meiotic progression defects in *Syce1* and *Syce2* mutants are unlikely to account for lack of HORMAD1/2 depletion from axes. Moreover, as noted above, HORMAD1/2 depletion still occurs in mutants that are competent to assemble at least some SC, but that have similar spermatogenic blocks as *Syce1−/−* and *Syce2−/−* [13],[15],[48],[50],[51].

Based on these considerations, and taking into account the observation that HORMAD depletion is highly specific for axes that have engaged in SC assembly, we suggest that the simplest interpretation of our findings is that the accumulation of SC components on AEs, on their own or in combination with other proteins, induces localized HORMAD depletion. How might this work?

SC formation is required for crossing over and efficient DSB repair in mice [11],[12],[14],[62],[63]. Hence, one could argue that SC formation promotes HORMAD1/2 depletion solely via promoting DSB repair. However, HORMADs are depleted from illegitimately synapsed axes in *Spo11−/−* and *Dmc1−/−* mutants (Figure 11, 13, S6 and S7), in which DSBs either do not form or are not repaired [2],[3],[52],[53]. Thus, progression of homologous recombination is not strictly required for HORMAD depletion from synapsed axes.

Nevertheless, we note that more HORMAD1 remains on synapsed axes in *Spo11−/−* and *Dmc1−/−* mutants than in WT, and HORMAD1 levels on unsynapsed axes also appear higher in these mutants (Figure 11, 13, S6 and S7). One possibility is that HORMAD1 somehow distinguishes aligned homologues from interactions between non-homologous chromosomes. Alternatively, it is possible that efficient HORMAD1 depletion may be partially dependent on normal execution of DSB repair. Precedent for such a dependency is found in *C. elegans*, where the timing and spatial organization of HORMA-domain protein depletion from chromosomes is intimately tied to recombination [37]. In mice, early steps in recombination are required for the robust formation of homologous SCs, so it is possible that the DSB repair process could support HORMAD1 depletion indirectly via promoting normal SC formation. Indeed, SYCP1 staining is relatively weak in *Dmc1−/−* and *Spo11−/−* mutants, most similar to the SYCP1 staining of freshly formed SCs in WT zygotene cells (Figure 11 and S6). Thus, non-homologous SCs might be qualitatively different from normal pachytene SCs (e.g., immature, or unstable). Moreover, we find a negative correlation between SYCP1 levels and residual HORMAD1/2 levels on synapsed axes in WT, which indicates that SC maturation might influence the efficiency of HORMAD1/2 depletion (Figure 5).

Finally, an alternative way to account for the correlation between recombination progression and the efficiency of HORMAD1 depletion is to propose that both SC formation and the production of late recombination intermediates promote HORMAD1 dissociation independently from each other. Although we can not exclude this possibility, behaviour of HORMADs on sex chromosomes argues against this idea. Even though DSBs are repaired on unsynapsed regions of sex chromosomes during late pachytene in WT cells (as judged by the disappearance of axis associated foci of RAD51 and RPA) [41],[45],[46], HORMAD1/2 levels nonetheless remain high on these unsynapsed axes during pachytene and diplotene (Figures 2, 3, 4). Hence, we infer that progression of DSB repair is not sufficient to trigger robust depletion of HORMADs from sex chromosomes.

In summary, we suggest that a simple interpretation of our data is that SC formation is required for depletion of HORMADs from axes, and that SC assembly can promote HORMAD1/2 depletion in the absence of the DSB repair process. Thus, we propose that the reciprocal distribution of SYCP1 and HORMADs on axes in normal meiosis is a consequence of HORMAD1/2 depletion from chromosome axes in response to SC formation. In contrast, DSB formation and repair are not absolutely required for depletion of HORMADs, although DSB repair steps downstream of SC formation may increase the efficiency of SC-promoted HORMAD1 depletion.

### Conserved relationship between PCH2/TRIP13 AAA-ATPases and HORMA-domain proteins

We show here that TRIP13 is required for depletion of HORMADs from synapsed axes (Figure 14). Because HORMAD1/2 depletion from SCs can occur in the presence of unrepaired DSBs in a *Dmc1−/−* mutant, we infer that the persistence of HORMADs on synapsed axes in *Trip13^hypo^* spermatocytes is unlikely to be a consequence of the delayed DSB repair in this mutant. Instead, it is more likely that TRIP13 promotes depletion of HORMADs from synapsed axes independently from DSB repair. TRIP13 activity could mediate depletion of HORMADs from axes in response to SC formation or, alternatively, TRIP13 could modify properties of SCs in a manner that promotes HORMAD1/2 depletion from axes. It is also possible that TRIP13 and SC act independently from each other such that neither is sufficient alone, but in combination they promote HORMAD1/2 depletion from axes (Figure S8).

Budding yeast Hop1 and the SC transverse filament protein, Zip1, exhibit reciprocal localization patterns along chromosome axes, and Pch2 is required for the depletion of Hop1 from Zip1-rich regions, which most likely represent fully synapsed axes [58]. It is not known whether Hop1 is depleted from chromosome axes in response to progression in DSB repair or synapsis formation *per se*. Nevertheless, the apparent similarity between the behaviour of HORMA-domain proteins in yeast and mouse suggests that Pch2/TRIP13 supports depletion of Hop1/HORMADs from synapsed chromosome axes in both organisms.

### What are the functions of HORMADs in early prophase, and why do they become depleted from synapsed chromosome axes?

The regulated depletion of HORMA-domain proteins from synapsed chromosomes is a striking feature of meiosis shared by budding yeast, rice, *C. elegans* and mouse [37],[58],[64],[65]. The conservation of this phenomenon suggests that removal of HORMA-domain proteins from synapsed axes may play an important role during meiosis. The cytological studies presented here do not allow us to definitively determine the functions of mammalian HORMADs. Nevertheless, our results in combination with published analyses of HORMA-domain proteins in other organisms allow us to speculate about the possible roles of HORMADs (Figure S8). We discuss here four possible functions that might no longer be needed, or that might need to be actively down-regulated, after SC formation.

#### DSB formation

First, HORMADs might promote efficient DSB formation similarly to budding yeast Hop1 and *C. elegans* HTP-3 [5],[35],[66]. The formation and repair of meiotic DSBs are strictly regulated to achieve proper homologue pairing and crossover formation. In particular, DSB repair must be directed toward homologues (IH bias) during meiosis. Thus, DSB formation is coordinated with establishment of the IH bias enforcing machinery. In budding yeast, DSB formation is promoted by axis component proteins Red1 and Hop1, which are also required for IH bias [5],[28]. We show here that mouse HORMADs are present on the developing axes upon which RAD51 foci form during leptotene/early zygotene (Figure S4). These results thus place HORMADs at the right time and place to potentially facilitate DSB formation. If HORMADs do play such a role, it is also interesting to note that, since homologous recombination in mice promotes homology search and homologue alignment, generation of new DSBs is most probably not needed after homology search is finished and SCs have formed. Thus, depletion of HORMADs from synapsed axes may contribute to down-regulation of DSB formation late in meiotic prophase.

#### Inhibiting promiscuous SC formation

Second, HORMADs could destabilize nascent SCs, thereby helping to ensure that mature, stable synapsis does not occur between inappropriate partners. Such a function has been inferred for HTP-1 in *C. elegans* [32],[33], so it is possible that mammalian HORMADs may have a similar role. However, relatively normal-looking SCs can form despite the persistence of high levels of HORMADs during zygotene and pachytene in the *Trip13^hypo^* mutant [61]. Thus, either HORMADs cannot inhibit SC formation between homologues, or TRIP13 is required together with HORMADs for SC destabilization. Another issue that argues against this function for HORMADs is that HTP-1 may not be as closely related to mouse HORMADs as other HORMA-domain proteins are. For example, HTP-1 is not depleted from synapsed axes during early stages of meiosis [37]; budding yeast Hop1 and *A. thaliana* ASY1 are necessary to make SCs rather than being needed to inhibit promiscuous SC formation [28],[31]; and HORMAD1/2 protein sequences are more similar to Hop1 and ASY1 than to *C. elegans* HORMA-domain proteins (Figure S2). Moreover, full homologous SC formation does not require DSB formation or recombination in *C. elegans* [67], unlike in yeast, mouse, or plants [2], [3], [68]–[71]. The different interplay between SC formation and recombination may thus have resulted in the evolution of different specialized functions for the *C. elegans* proteins that are not conserved in other organisms.

#### Inter-homologue bias and regulation of the progression of recombination

Third, HORMADs could be required for meiotic IH bias, in which recombinational repair of DSBs from the homologue is promoted at the expense of repair from the sister chromatid, thereby ensuring that DSBs promote the search for homology, homologue alignment, and crossing over. *S. cerevisiae* Hop1 and meiotic HORMA-domain proteins in *Arabidopsis* and *C. elegans* are required for IH bias [5], [25], [29], [32]–[34],[36]. This function may thus be conserved for HORMAD1 and -2 in mice. SCs are not required for homologue alignment in yeast and mammals [11],[14],[62],[72],[73], rather, homologue alignment appears to be essential for correct and complete SC formation [2]–[4],[52],[53],[70],[74],[75], and indeed, complete formation of the SC may mark the end of the homologue alignment process. Thus, we would argue that IH bias is no longer required once homologues have aligned and SCs have formed. At this point, cells have three important tasks: crossover-designated strand invasion events must be fully processed into mature crossovers and chiasmata; all remaining DSBs that have engaged with homologous DNA sequences must be repaired without reciprocal exchange (i.e., as noncrossovers); and, importantly, DSBs that did not find a partner on the homologue must be repaired from the sister chromatid in order to avoid either permanent arrest/apoptosis or entry into meiotic divisions with unrepaired DNA damage. Depletion of HORMADs from synapsed AEs might couple completion of homologue alignment and SC formation with down-regulation of the homology search and IH bias, and it may allow inter-sister repair of DSBs that might have failed to engage with homologous chromosomes during the homology search process.

The mechanism of IH bias is not yet well understood in any organism. The roles of axis associated HORMA-domain proteins in this process are thus not clear, but it has been suggested that they function in part by interfering with at least some kinds of DSB repair, e.g., DSB repair specifically from sister chromatids [5],[29]. In this context, it is interesting to note that our findings suggest that persistence of HORMADs on synapsed axes might cause delayed DSB repair in mice. Specifically, we find that *Trip13^hypo^* mutants, which are known to have delays in completing DSB repair as judged by persistence of RAD51 foci and other recombination markers [61], also are defective for HORMAD1/2depletion from synapsed axes (Figure 14). We do not yet know if this correlation reflects a causal relationship between persistence of HORMA-domain proteins on synapsed chromosome axes and delayed DSB repair, but we do note that yeast *pch2* mutants reveal a similar correlation [58]. Deletion of Pch2 in yeast affects both inter-homologue and inter-sister repair [58]. Yet, it is unclear if both pathways were affected in the *Trip13^hypo^* mutant [61]. It is tempting to speculate that the failure to displace HORMADs (or Hop1) from synapsed axes contributes directly to the delay in progression of homologous recombination, and that the normal TRIP13-dependent depletion of HORMADs from synapsed chromosome axes may facilitate the rapid repair of DSBs that are not committed to inter-homologue repair. Importantly, if the above hypothesis is correct, it further implies that HORMA-domain proteins may have a previously unrecognised role in regulating the progression of DSB repair from all homologous templates, not just their proposed role in inhibiting DSB repair from sister chromatids.

Nonetheless, even if HORMADs do serve this function, their depletion from axes is unlikely to be the only way to down-regulate IH bias because HORMADs accumulate and persist on the unsynapsed regions of sex chromosomes in pachytene spermatocytes, yet DSBs in these regions are eventually repaired despite the presence of high HORMAD1/2 levels. Thus, it may be that HORMADs and/or IH bias can be inactivated by some other means, such as changes in post-translational modifications. In budding yeast, phosphorylation of Hop1 by Mec1 and Tel1 is required for IH bias but does not affect Hop1 localization [25]. We found that a significant proportion of chromatin-associated HORMAD1 and -2 are phosphorylated during prophase (unpublished results of V. Boonsanay and A. Tóth), and both HORMADs contain putative ATM/ATR phosphorylation sites (data not shown), so a similar regulatory mechanism could exist in mammals. Alternatively, the DSB repair machinery might change during progression through pachytene, thereby allowing DSB repair on sex chromosomes in the presence of HORMADs. Indeed, response to irradiation induced DSBs seems to change as mouse spermatocytes progress from mid to late pachytene [76]. Precedent for a change in the mode of DSB repair during pachytene has also been reported in *C. elegans*, where competence to convert DSBs into inter-homologue COs is lost in late pachytene [77].

#### ATR, MSUC, and meiotic checkpoints

Finally, HORMADs might be involved in the mid pachytene checkpoint. Restriction of ATR activity/γH2AX to sex chromosomes by full SC formation on autosomes is believed to promote efficient silencing of sex chromosomes, which is assumed to be a prerequisite for progression beyond the mid pachytene checkpoint [17]–[19],[22]. HORMADs and known MSUC-promoting proteins, such as ATR, BRCA1 and TOPBP1, are all depleted from synapsed chromosome regions and accumulate on unsynapsed regions of sex chromosomes during pachytene (this study and [18],[57],[78],[79]). Furthermore, we find that HORMAD1/2 hyper-accumulation on unsynapsed axes is correlated with ATR activity/γH2AX accumulation in *Spo11−/−* cells (Figure 13). These observations raise the possibility that there may exist a functional interaction between ATR/MSUC and HORMADs.

Interestingly, phosphorylation of budding yeast Hop1 by yeast ATR/ATM homologs, Mec1/Tel1, is required for the activity of the meiotic prophase checkpoint that monitors DSB repair [25]. Hop1 was proposed to be an adaptor protein that facilitates activation of the DNA-damage effector kinase Mek1 in response to Mec1/Tel1 activation [25]. Therefore, it is tempting to speculate that mouse HORMADs could collaborate with ATR in promoting MSUC. If this hypothesis was correct, it would imply that depletion of HORMADs from synapsed autosomes could be one sufficient mechanism to restrict MSUC to the sex chromosomes, thereby satisfying the mid pachytene checkpoint.

Nevertheless, we should point out that even if HORMADs are involved in MSUC, other controls next to HORMAD1/2 depletion likely exist for turning off the mid pachytene checkpoint [61]. Despite abnormally persisting low levels of γH2AX on synapsed chromatin in the *Trip13^hypo^* mutant, γH2AX accumulates to higher levels on chromatin surrounding unsynapsed sex chromosomes than on the chromatin of synapsed autosomes [61]. This indicates that SC formation can down-regulate ATR activity independently from HORMADs (Figure S8).

### Possible functions late in meiosis, and differences between HORMAD1 and -2

The continued presence of HORMADs on chromosomes after diplotene suggests the possibility of a role in meiosis after the completion of recombination. *C. elegans* HTP-1 is required for maintenance of centromeric sister chromatid cohesion during the first meiotic division [37]. We found that HORMADs localize near centromeres in metaphase I spermatocytes (Figure 6.), suggesting that HORMADs may also be involved in this essential characteristic of meiosis-specific chromosome behaviour in mice.

The behaviours of the two HORMADs are similar, but not identical, so it is not yet possible to determine if they are involved in identical or only partially overlapping processes. For example, HORMAD2 is more likely than HORMAD1 to show preferential accumulation within pseudo-sex bodies (Figure 13 and S7). We also saw differences in localization during WT diplotene in males, with HORMAD2 more highly enriched on the sex chromosomes than on the desynapsing autosomes, as compared with HORMAD1 (Figure 2, 3 and 4). These differences may be a reflection of different abundance of the two proteins (their relative amounts are not yet known), or may reflect a genuine difference in the relationship between MSUC pathway components and the two HORMADs. There are also differences in the extent of overlap between forming SCs and the two HORMADs during zygotene, with HORMAD2 tending to spread more into synapsed regions than HORMAD1 (Figure 4 and 5). This difference may indicate that the two proteins are depleted from synapsed axes with different kinetics, and/or that they respond to different SC-associated processes.

The resolution of these questions will require the generation of HORMAD mutant mice, which is the next logical step to precisely determine the meiotic functions of HORMADs in mammals.

## Materials and Methods

### Animals

Female NZW rabbits and female Hartley guinea pigs were used for immunization experiments. For expression analysis, immunofluorescence and immunoblot wild type (WT) testis tissue was isolated from C57BL/6JOlaHsd mice and WT embryonic ovaries were obtained from NMRI mice. For staging embryonic development the day of detection of a vaginal plug was marked as 0.5 days post coitum (dpc). Analysed null mutant mice strains (*Smc1β−/−*, *Syce1−/−*, *Syce2−/−*, *Rec8−/−*, *Dmc1−/−*, *Spo11−/−*, *Atm−/−*) have been described previously [3],[11],[14],[48],[51],[53],[56]. The commercially available ES clone with the gene trap-disrupted allele of Trip13 was described previously [61]. The *Trip13* ^RRB047/RRB047^ strain we studied was generated by I. Roig, M. Jasin and S. Keeney (unpublished). This mouse line carries the same mutation as the previously described *Trip13* mutant line but the line was generated independently from the previous study [61]. Experimental animals were compared with controls from the same litter (when possible) or from other litters from the same mating. All animals were used and maintained according to regulations provided by the animal ethical committee of the Technische Universität Dresden.

### RNA-isolation and RT-PCR

Total RNA was isolated from fresh adult mouse testis tissue and frozen embryonic gonads using the RNeasy Mini Kit (Qiagen). Mouse total RNA samples from different mouse somatic tissues were purchased via Ambion (liver, brain, thymus, heart, lung, spleen and kidney, Cat#7800) and Zyagen (mammary gland, pancreas, placenta, salivary gland, skeletal muscle, skin, small intestine, spinal cord, tongue and uterus, Cat#MR-010). One or half micrograms of total RNAs were reverse transcribed using Superscript III (Cat#18080-044, Invitrogen) and oligo dT (20) primers. In no-RT controls the reaction mixture contained water instead of reverse transcriptase. RT-PCR was performed with gene specific primers: 5′- TGTTTGTCACCTACACTCAGG-3′ and 5′-GTAAGGAAGAAGAAACTATGC-3′ for *Hormad1*, 5′- CCTGCAAGTTACAGACAGATA-3′ and 5′-AACCTGTGAGTTGGAATCCT-3′ for *Hormad2*. The primes for amplifying *Sycp3*, *Mvh*, *Xist*, *S9* and *S12* as controls were described previously [80]. The cycling conditions were: 94°C 3 min; 94°C 30 s, 54°C 30 s, and 72°C 25 s for 30 cycles; and 72°C 7 min. RNA-Isolation from FACS sorted ovarian cells and RT-PCR on these templates were performed as described previously [80].

### Antibody generation

HORMAD1 and -2 are most similar in their HORMA-domain containing N-terminal region (Figure S2B). Their C-terminus differs considerably. To avoid cross-reactivity, we raised antibodies against the less conserved C-terminal domain of HORMADs. The cDNA fragments encoding for the C-terminal 142 amino-acids of Hormad1 (H1C) and the C-terminal 72 amino-acids of Hormad2 (H2C) were sub cloned into the *Escherichia coli* expression vectors pDEST17 (Cat#11803012, Invitrogen) and pDEST15 (Cat#11802014, Invitrogen), respectively. H1C was expressed in fusion with N-terminal 6xHis-tag and purified on Ni Sepharose (Cat#17-5318-01, Amersham, GE Healthcare). H2C was expressed in fusion with an N-terminal GST-tag and purified on Glutathione Sepharose (Cat# 17-5132-01, Amersham, GE Healthcare). One guinea pig and two rabbits were immunized with each of the two recombinant proteins (H1C and H2C) [81]. Polyclonal antibodies were affinity purified on antigen coupled Sepharose Beads (Cat# 17-0906-01, Amersham, GE Healthcare). Specificity of affinity purified anti-Hormad1 (rabbit polyclonal AB209 and AB153 and guinea pig polyclonal AB146) and anti-Hormad2 (rabbit polyclonal AB205 and AB211 and guinea pig polyclonal AB104) antibodies were tested by immunoblot analysis of testis extracts. Anti-HORMAD1 and anti-HORMAD2 antibodies recognise different proteins in testis extracts (Figure S3). All of our affinity purified anti-HORMAD1 antibodies recognise a protein, which is approximately 50 kDa based on its electrophoretic mobility. The anti-HORMAD2 antibodies recognise a protein that migrates as a 40 kDa protein during SDS poly-acryl amide electrophoresis. The estimated masses of the recognised proteins are consistent with the calculated molecular mass of HORMAD1 (43 kDa) and HORMAD2 (35 kDa). AB205, 209 and 211 recognize additional proteins other than HORMAD1 and 2 in immunoblots (Figure S3). These cross-reactive proteins are enriched in the detergent soluble fraction of testicular cells as opposed to HORMAD proteins that are enriched in the detergent insoluble (crude chromatin) fractions of testis extracts.

### Preparation of testis protein extracts

Testis tissue from 20 days old C57BL/6 mice were minced with a surgical blade and homogenized by a loose-Dounce homogenizer with about 30 strokes in ten times volume (w/v) of PBS pH 7.4 containing 1 mM EDTA (Cat#E5134, Sigma), 1× Complete EDTA free protease inhibitor cocktail (Cat#11873580001, Roche), 25 mM b-Glycerophosphate (Cat#35675, Merck), 10 mM Na4P2O7 (Cat#71515, Sigma) , 50 mM NaF (Cat#P0759S, NEB), 2 mM Na3VO4 (Cat#P0758L, NEB,) and 1 mM PMSF (Cat#10236608001, Roche). The cell suspension was filtered through a 40 µm sieve (BD BioScience, San Jose, CA) and centrifuged for one minute at 960 rcf. The pellet was resuspended in 1 ml of RSB-G with 0.25% NP-40 (Cat#74385, Sigma,), 1 mM DTT (Cat#D9779, Sigma,) containing protease and phosphatase inhibitors listed above and then centrifuged at 10,000 rcf for one minute [82]. The supernatant was collected and used for western blot analysis (NP-40 soluble testis fraction). The pellet was washed once with RSB-G (without NP-40) and once with RIPA buffer containing protease and phosphatase inhibitors listed above. Following one minute centrifugation with 10000 rcf a pellet was obtained, which was dissolved by five minutes of boiling in SDS loading buffer and used for immunoblot analysis of HORMAD antibodies (detergent insoluble testis fraction).

### SDS-PAGE and immunoblotting

Proteins from testis extracts were separated on 13% SDS poly-acryl amide gels and blotted onto PVDF membrane (Cat#P2938, Sigma). The membranes were incubated with antibodies at 1∶500 (AB209), 1∶1000 (AB153), 1∶500 (AB146), 1∶200 (AB205), 1∶2000 (AB211) and at 1∶500 (AB104) dilutions in Western-incubation buffer (5% milk, Tris-buffered saline plus 0.05% Tween 20). Membrane bound primary antibodies were detected by 1∶10000 diluted HRP-coupled Goat Anti-Rabbit IgG or Goat Anti-Guinea Pig (Cat#111-035-144 and Cat#106-035-003, Jackson ImmunoResearch) antibodies using the Immobilon Western Chemiluminescent HRP Substrate (Cat# WBKLS0100, Millipore).

### Immunofluorescence (IF)

“Standard” nuclear surface spreads of spermatocytes and oocytes were prepared either as described previously or according to a modified protocol [83]. Briefly, cell suspensions were prepared in PBS by vigorous pipetting of the gonads. In the modified protocol, slides were covered with a thin layer of 0.25% NP-40 (Sigma). Cell suspensions of fresh or frozen testis or ovaries in PBS pH 7.2 (one third volume of NP-40) were pipetted onto the NP-40 surface and incubated for no longer than 2 min before adding drop by drop three times the NP-40 volume of S-fix fixative (1% paraformaldehyde, 10 mM sodium borate buffer pH 9.2). Samples were incubated for two hours at room temperature in a humid chamber. Following fast drying under a hood, the slides were washed two times for one minute with 0.4% Agepon (AgfaPhoto) and another three times for one minute with water. Slides were used immediately or kept at 4°C in PBS pH 7.4 until IF staining. The “disrupted” nuclear spreads were prepared by extending incubation time of cells in 0.25% NP-40 up to 20 min before addition of S-fix.

For cryo-sections, adult testes were fixed in 2% formaldehyde in PBS pH 7.4, 0.1% Triton X-100 at room temperature for 20 min. Following fixation, testis was placed into 30% sucrose overnight at 4°C and then frozen on dry ice in “O.C.T.” (Sakura Finetek Europe). 8 µm thick sections were cut and dried onto slides followed by five minute fixation by 2% formaldehyde in PBS pH 7.4, 0.1% Triton X-100. The sections were washed in PBS pH7.4 and immediately used for IF staining.

Before immunostaining surface spreads or cryo-sections, samples were blocked with blocking buffer [2% BSA (Cat# A2153, Sigma), 10% goat serum (Cat# ab7481, Abcam), 0.1% Triton X-100 in PBS pH 7.4] for 30 min. Primary antibodies diluted in blocking buffer were applied to samples for three hours or overnight at 37°C in a humid chamber. Slides were washed three times with PBS and incubated with secondary antibodies for 1 h, and finally mounted in Vectashield mounting medium with DAPI (Cat#H-1200, Linaris).

Primary antibodies used in this study were as follows: rabbit anti-HORMAD1 AB209 (1∶2000) and AB153 (1∶1000), guinea pig anti-HORMAD1 AB146 (1∶500), rabbit anti-HORMAD2 AB205 (1∶2000) and AB 211 (1∶3000), guinea pig anti-HORMAD2 AB104 (1∶500), monoclonal mouse anti-SYCP3 II52F10 (1∶100, a gift from R. Jessberger) [84], rabbit anti-SYCP3 (1∶1000, Cat#ab15092, Abcam, Cambridge, MA), mouse anti γ-H2AX (1∶3000, Cat#05-636, Upstate/Millipore), rabbit anti-SYCP1 (1∶1000, Cat#ab 15090, Abcam), rabbit anti-hRPA70 (1∶500, a gift from E. Marcon) [46], rabbit anti-hH1t (1∶1000, a gift from E. Marcon) [85], guinea pig anti H1t (1∶5000, a gift from M.A. Handel) [86], rabbit anti-TOPBP1 (1∶1000, a gift from J. Chen) [87], rabbit anti-TRF1 #644 (1∶2000, a gift from T. de Lange) , human anti-Centromere protein (1∶1000, Cat#15-235, Antibodies Inc.). Goat secondary antibodies conjugated with either Alexa Fluor 488, 568 or 647 (Cat# A11034, A11036, A21245, A11073, A11075, A21450, A21090, A11031, A11029, A21236, Molecular Probes/Invitrogen) were used at a dilution of 1∶600. Donkey secondary antibodies conjugated with DyLight488, 594 or 649 (Jackson ImmunoResearch Europe Ltd.) were used at 1∶300 dilutions. Fluorescence was visualized with Zeiss Axiophot fluorescence microscope.

To stain HORMADs on cryo-sections we only used AB153 and AB104, two antibodies that are highly specific to HORMAD1 and -2, respectively. In nuclear surface spreads of spermatocytes, where most of the detergent soluble cell material is removed, all antibodies raised against the same antigen (H1C or H2C) showed similar staining patterns.

To conclude on the pattern of fluorescence staining for various proteins, staining patterns were assessed by eye under the microscope in at least 100 spread nuclei. As a second step, at least 30 nuclei of particular meiotic stages were first identified based on the localization pattern of SYCP3 AE component, then imaged at appropriate wavelengths to determine the pattern of co-stained proteins such as HORMADs, TOPBP1 or SYCP1, etc. At least two independent sets of nuclear spreads were examined from each mutant. Apart from the *Rec8−/−* mutant, we examined nuclear spreads from at least two different animals. Unless indicated differently, the panels shown in the figures were the exclusive or predominant patterns seen.

### Quantification of IF signal

To assess changes in chromosome associated staining of HORMAD1, HORMAD2 and SYCP1 we quantified IF signals specific to these proteins along synapsed and unsynapsed chromosome axes in at least 15 randomly picked nuclei of WT and *Dmc1−/−* mutants. To compare IF signal levels in WT and *Dmc1−/−* mutant we prepared nuclear spreads parallel from mutant and control animals. Spread nuclei were co-stained with antibodies recognising SYCP3, SYCP1 and either one of the HORMADs. Mutant and WT spreads were stained at the same time with the same mixes of antibodies. Imaging of the cells in each experiment were carried out in the same day with the same microscope and camera settings and TetraSpeck Fluorescent Microspheres Size Kit, T14792 Molecular Probes, Invitrogen were used to control for possible changes in illumination during the course of imaging. Measurement of IF signal was carried out with the help of Adobe Photoshop CS4 Extended version. SYCP3/axis staining was used to select synapsed and unsynapsed chromosome axes that belong to a single chromosome. In the second step, we measured total IF signal intensity of HORMADs and/or SYCP1 in identical-sized rectangles that were placed over straight stretches of the selected synapsed and unsynapsed chromosome axes. Signal intensities were also measured in four regions around examined chromosomes in each nucleus in order to estimate the background. Signal intensity values shown in Figure 5 and 11 are background corrected. Whenever it was possible we measured signal intensities on at least two chromosomes in each nucleus. Statistical analysis was performed with GraphPad Prism 5. For statistical analysis Wilcoxon signed-rank test was used when paired signal intensities on unsynapsed and synapsed axes of WT zygotene chromosomes were compared. For the comparison of independent samples two-tailed non-parametric Wilcoxon–Mann–Whitney two-sample rank-sum test was used. We acknowledge that there are clear limitations to quantification of IF signal on nuclear spreads. Due to the nature of the spreading methodology variable amounts of soluble and axis associated proteins are removed from each spread nucleus. Therefore, there is considerable variation in IF signal intensities and background levels within the same sample preparation and in between sample preparations. Hence, we do not think that these measurements can be used to accurately determine fold changes in protein levels on chromosome axes. Nevertheless, we think that the presented quantifications are suitable to illustrate tendencies in the data. They also reconfirm conclusions we drew from the observation of larger number of cells in larger number of experiments.

### Phylogenetic analyses

HORMAD1 and HORMAD2 orthologs were identified by blastp alignments of HORMAD1 and HORMAD2 sequences of Genbank protein database. Accession numbers of used sequences are shown in supplementary table S1. Protein sequence alignments were prepared with ClustalW software using the entire amino-acid sequences. The tree was constructed in MEGA4 software [88]. The neighbour-joining method with Poisson correction was used. The reliability of internal branches was assessed by using 500 bootstrap replicates, and sites with gaps were ignored in this analysis.

## Supporting Information

Figure S1 *Hormad1* and *Hormad2* are specifically expressed in male and female meiotic germ cells. RT-PCR was used to measure expression of *Hormad1*, *-2*, “house-keeping” genes *(S9* and *S12)*, a germ cell marker *(Mvh)*, a somatic cell marker in females (*Xist*) and a meiosis marker (*Sycp3*). (A) Expression of *Hormad1* and *Hormad2* is specific to gonadal tissue. cDNAs were prepared from four RNA mixtures that contained RNAs from testis and from non-gonadal tissues in different ratios. (1) Somatic tissue mix: 1 µg of total RNA made up by mixing 59 ng of total RNA from 17 somatic tissues. (2) Testis: 59 ng total RNA from adult testis. (3) Somatic+testis mix: 1 µg of total RNA made up by mixing 59 ng total RNA from testis with 941 ng of somatic tissue mix. (4) Somatic+5xtestis: 1 µg of total RNA made up by mixing 295 ng total RNA from testis with 705 ng of somatic tissue mix. (5) noRT control with somatic+testis mix. The 17 analysed somatic tissues are: liver, brain, thymus, heart, lung, spleen, kidney, mammary gland, pancreas, placenta, salivary gland, skeletal muscle, skin, small intestine, spinal cord, tongue, uterus. *Hormad1*- and *Hormad2*- specific PCR-products were amplified only from templates that contained testis cDNA. (B) RT-PCRs were performed on cDNAs prepared from FACS sorted mixed ovarian cells, ovarian somatic cells and germ cells collected at 16.5 days post coitum (dpc) (see Materials and Methods). Purity of cell populations is assessed by RT-PCRs specific to *Xist* and *Mvh* marker genes. *Hormad1*, *Hormad2* and *Mvh* are exclusively expressed in ovarian germ cells. (C) RT-PCRs were performed on cDNAs prepared from testis at the indicated ages. Expression of both *Hormad1* and -*2* is strongly up-regulated as the first wave of germ cells enters meiosis after 7 days post partum (dpp) and reaches pachytene at 15dpp. (D) RT-PCRs were performed on cDNAs prepared from ovaries at the indicated times post fertilization. Expression of *Hormad1*, *Hormad2* and *Sycp3* is up-regulated as female germ cells enter meiosis between 12.5 and 14.5 dpc. *Hormad1* expression levels peak at 16.5 dpc, when germ cells start to enter pachytene. *Hormad2* expression peaks at 14.5 dpc, when most germ cells are in leptotene or early zygotene.
(1.08 MB TIF)

Figure S2 HORMAD1 and HORMAD2 are related to Hop1-like HORMA-domain proteins. (A) Phylogenetic tree of HORMA-domain containing proteins. The meiosis-specific Hop1 branch of HORMA-domain proteins is marked in green. Numbers are bootstrap values (see Materials and Methods). The full length amino acid sequences were used for the analysis. Accession number of each protein is presented in Table S1. (B) Alignment of *Mus musculus* HORMAD1 and HORMAD2, *Arabidopsis thaliana* ASY1, *Saccharomyces cerevisiae* Hop1 and *Caenorhabditis elegans* HIM-3 proteins. Black: Identical amino acids. Gray: Similar amino acids. The conserved HORMA-domain region is underlined [24].
(1.57 MB TIF)

Figure S3 No cross-reactivity is observed between anti-HORMAD1 and anti HORMAD2 antibodies on immunoblots (IB). Detergent insoluble (I) and NP-40 soluble (S) fractions of 20 dpp mouse testis extracts were prepared as described in Materials and Methods. Following SDS-PAGE, immunoblot analysis was used to determine the molecular weight of proteins recognized by affinity-purified antibodies raised against the C-terminus of HORMAD1(αH1C) and HORMAD2 (αH2C). Fractionation of testis extracts was controlled by detection of HISTONE3 on all blot membranes. αH1C and αH2C antibodies recognize different proteins. All three αH1C antibodies (rabbit polyclonal AB209 and AB153 and guinea pig polyclonal AB146) recognized a protein which migrates slightly slower than what is predicted for HORMAD1. The additional, slower migrating protein detected by all of our αH1C antibodies in the detergent-insoluble fraction (*) is a phosphorylated form of HORMAD1 (our unpublished results). All αH2C antibodies (rabbit polyclonal AB205 and AB211 and guinea pig polyclonal AB104) recognized a protein which migrates slightly slower than what is predicted for HORMAD2.
(0.43 MB TIF)

Figure S4 RAD51 foci closely associate with HORMAD1- and HORMAD2-associated axes during leptotene/early zygotene. SYCP3, RAD51, and either HORMAD1 (A) or HORMAD2 (B) were detected on nuclear spreads of leptotene/early zygotene spermatocytes. RAD51 foci are closely associated with forming axes decorated with HORMAD1 and HORMAD2. Bars, 10 µm.
(1.30 MB TIF)

Figure S5 Localization of HORMAD1 and -2 to late leptotene and early zygotene chromosome axes is independent of DSB formation and ATM. Indicated proteins were detected by IF on nuclear surface spreads of WT (A), *Spo11−/−* (B) and *Atm−/−* (C) spermatocytes. Images were taken with the same camera settings to facilitate comparison of protein levels. Bars, 10 µm. (A) HORMAD1 and -2 appear on developing chromosome axes during leptotene in WT cells. In response to DSB formation ATM kinase promotes accumulation of γ-H2AX on chromatin at the time of axis formation. (B,C) HORMAD1 and -2 accumulate on the developing chromosome axes during leptotene in the absence of DSBs and ATM kinase activity (n = 100 cells). Accumulation of γ-H2AX on chromatin requires both DSBs and ATM during this early stage of prophase.
(4.23 MB TIF)

Figure S6 HORMAD1 levels on synapsed and unsynapsed axes are higher in *Spo11−/−* spermatocytes than in WT zygotene cells. SYCP3, SYCP1, and HORMAD1 were detected by IF on nuclear spreads that were prepared in parallel from WT (A and B) and *Spo11−/−* (C) testes. HORMAD1 and SYCP1 staining were compared on matched exposures of 30 randomly picked nuclei in two independent experiments. Representative images of WT zygotene (A), WT pachytene (B) and *Spo11−/−* (C) spermatocytes are shown. SYCP1 levels on non-homologously synapsed axes in the *Spo11−/−* mutant (C) are comparable to levels on homologously synapsed axes in WT zygotene cells (A) but are much lower than in WT pachytene cells. HORMAD1 signal on both unsynapsed and synapsed axes is higher in *Spo11−/−* cells than for the corresponding synaptic configurations in WT zygotene cells. Nevertheless, HORMAD1 signal in the mutant cells is substantially reduced on synapsed axes as compared to unsynapsed axes (n = 100 cells examined). The asterisk marks the sex chromosomes in the WT pachytene cell (A middle row). Examples are indicated of synapsed (arrows) and unsynapsed (arrowheads) axes. Bars, 10 µm.
(1.37 MB TIF)

Figure S7 Localization of HORMAD1 and HORMAD2 in relation to TOPBP1. SYCP3, TOPBP1, and either HORMAD1 or HORMAD2 were detected by IF on nuclear spreads of spermatocytes from WT or *Spo11−/−* mutant testes. Bars, 10 µm. (A) Both HORMADs and TOPBP1 decorate unsynapsed axes in zygotene spermatocytes. Note that whereas HORMAD1 and -2 staining appears relatively continuous along unsynapsed chromosome axes, TOPBP1 instead forms dot-like foci. (B,C) In *Spo11−/−* spermatocytes, HORMADs preferentially localize to unsynapsed chromosome axes both within and outside of TOPBP1-rich regions, which correspond to pseudo-sex bodies [15]. In 9 out of 49 (18%) and 36 out of 55 (65%) “pachytene-like” cells (i.e., cells with locally restricted TOPBP1 accumulation (pseudo-sex body) and extensive synapsis), HORMAD1 and HORMAD2 hyper-accumulate in TOPBP1 rich regions, respectively. In the top rows of B and C, cells are shown in which HORMAD levels are comparable on unsynapsed axes inside and outside of TOPBP1-rich regions. In the bottom rows of B and C, cells are shown in which HORMADs hyper-accumulate within TOPBP1-rich regions. Examples are indicated of synapsed (arrows) and unsynapsed (arrowheads) axes. Bars, 10 µm.
(2.44 MB TIF)

Figure S8 Speculative working model for the functions of HORMADs during male meiosis. Open block-arrows represent processes, flat-end red arrows represent inhibition (direct or indirect) and red arrows represent activation (direct or indirect). Possible functions of axis-associated HORMADs may include: promoting DSB formation, inhibiting inter-sister repair of DSBs, inhibiting promiscuous SC formation, and collaborating with ATR in promoting MSUC (see text for details). Inhibition of inter-sister repair permits the use of resected DSB ends for homology search. Stable strand invasion of DSB ends into homologous DNA sequences and chromosome alignment promotes legitimate SC formation. In turn, SC facilitates repair of DSBs as crossovers or noncrossovers in mammals [10]. SC also promotes depletion of HORMADs from chromosome axes in collaboration with TRIP13 (SC and TRIP13 could act independently or in the same pathway). Because homology searching is no longer necessary after SC formation, it is plausible that synapsis is accompanied by down-regulation of DSB formation and of inhibition of inter-sister DSB repair. HORMAD depletion from synapsed axes may help to accomplish this down-regulation. Depletion of HORMADs from synapsed autosomes may be one mechanism to restrict MSUC to unsynapsed sex chromosomes, thereby promoting progression past the mid pachytene checkpoint in males. Even if HORMADs promote MSUC, SC formation is able to inhibit ATR activity independently from the depletion of HORMADs from chromosome axes: despite abnormal persistence of low levels of γ-H2AX along synapsed autosomes in *Trip13^hypo^* pachytene cells, γ-H2AX preferentially accumulates on unsynapsed sex chromosomes [61].
(0.15 MB TIF)

Table S1 Genbank accession numbers of HORMA-domain proteins used for phylogenetic comparison in Figure S2.
(0.22 MB TIF)
