# Supplementary figures and images for: Mouse HORMAD1 and HORMAD2, Two Conserved Meiotic Chromosomal Proteins, Are Depleted from Synapsed Chromosome Axes with the Help of TRIP13 AAA-ATPase
Source: PLoS Genet. 2009 Oct 23;5(10):e1000702. doi: 10.1371/journal.pgen.1000702 (PMC2758600; doi:10.1371/journal.pgen.1000702)

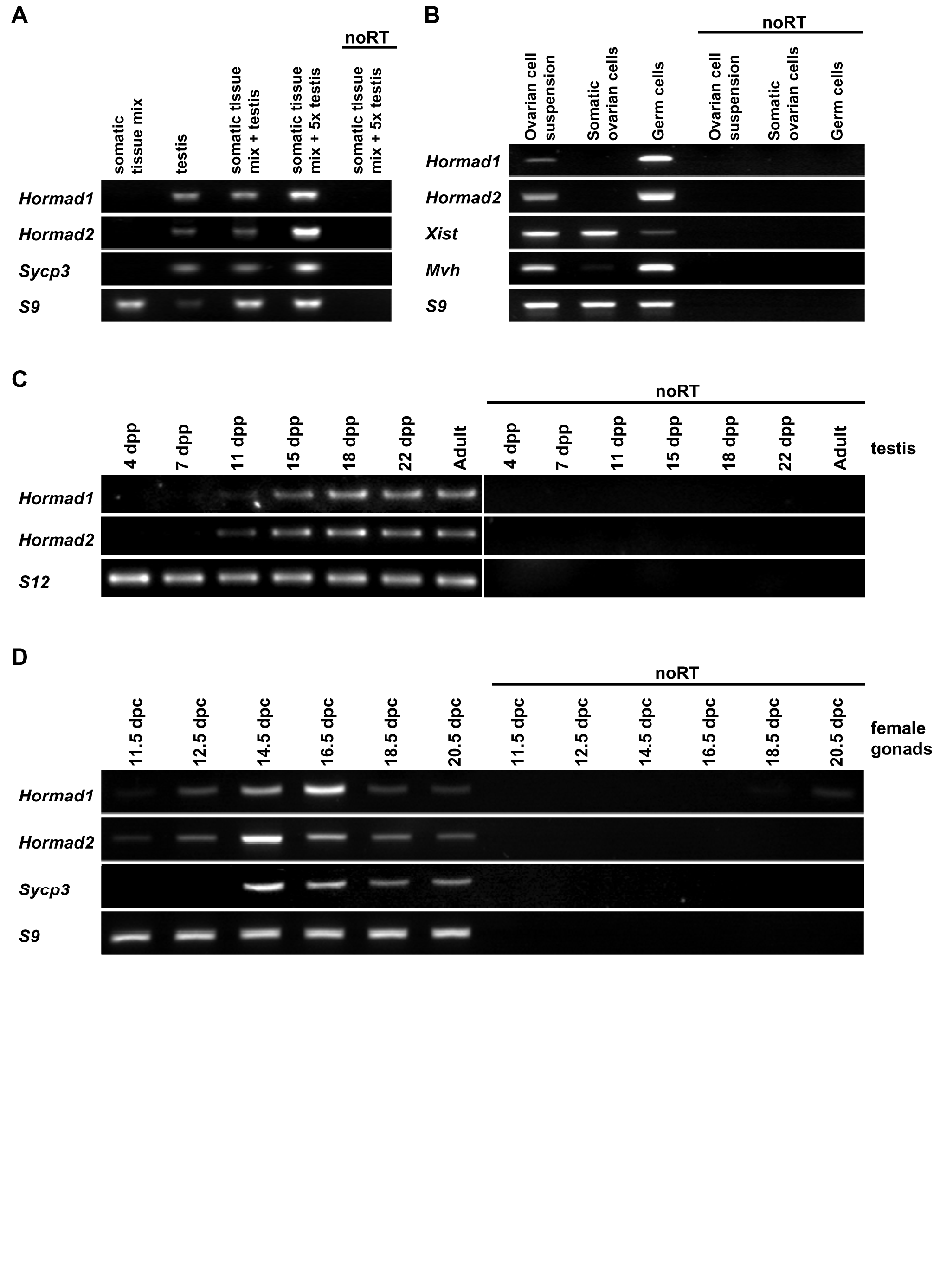

Supplement: Figure S1 — Hormad1 and Hormad2 are specifically expressed in male and female meiotic germ cells. RT-PCR was used to measure expression of Hormad1, -2, “house-keeping” genes (S9 and S12), a germ cell marker (Mvh), a somatic cell marker in females (Xist) and a meiosis marker (Sycp3). (A) Expression of Hormad1 and Hormad2 is specific to gonadal tissue. cDNAs were prepared from four RNA mixtures that contained RNAs from testis and from non-gonadal tissues in different ratios. (1) Somatic tissue mix: 1 µg of total RNA made up by mixing 59 ng of total RNA from 17 somatic tissues. (2) Testis: 59 ng total RNA from adult testis. (3) Somatic+testis mix: 1 µg of total RNA made up by mixing 59 ng total RNA from testis with 941 ng of somatic tissue mix. (4) Somatic+5xtestis: 1 µg of total RNA made up by mixing 295 ng total RNA from testis with 705 ng of somatic tissue mix. (5) noRT control with somatic+testis mix. The 17 analysed somatic tissues are: liver, brain, thymus, heart, lung, spleen, kidney, mammary gland, pancreas, placenta, salivary gland, skeletal muscle, skin, small intestine, spinal cord, tongue, uterus. Hormad1- and Hormad2- specific PCR-products were amplified only from templates that contained testis cDNA. (B) RT-PCRs were performed on cDNAs prepared from FACS sorted mixed ovarian cells, ovarian somatic cells and germ cells collected at 16.5 days post coitum (dpc) (see Materials and Methods). Purity of cell populations is assessed by RT-PCRs specific to Xist and Mvh marker genes. Hormad1, Hormad2 and Mvh are exclusively expressed in ovarian germ cells. (C) RT-PCRs were performed on cDNAs prepared from testis at the indicated ages. Expression of both Hormad1 and -2 is strongly up-regulated as the first wave of germ cells enters meiosis after 7 days post partum (dpp) and reaches pachytene at 15dpp. (D) RT-PCRs were performed on cDNAs prepared from ovaries at the indicated times post fertilization. Expression of Hormad1, Hormad2 and Sycp3 is up-regulated as female germ cells [file pgen.1000702.s001.tif]

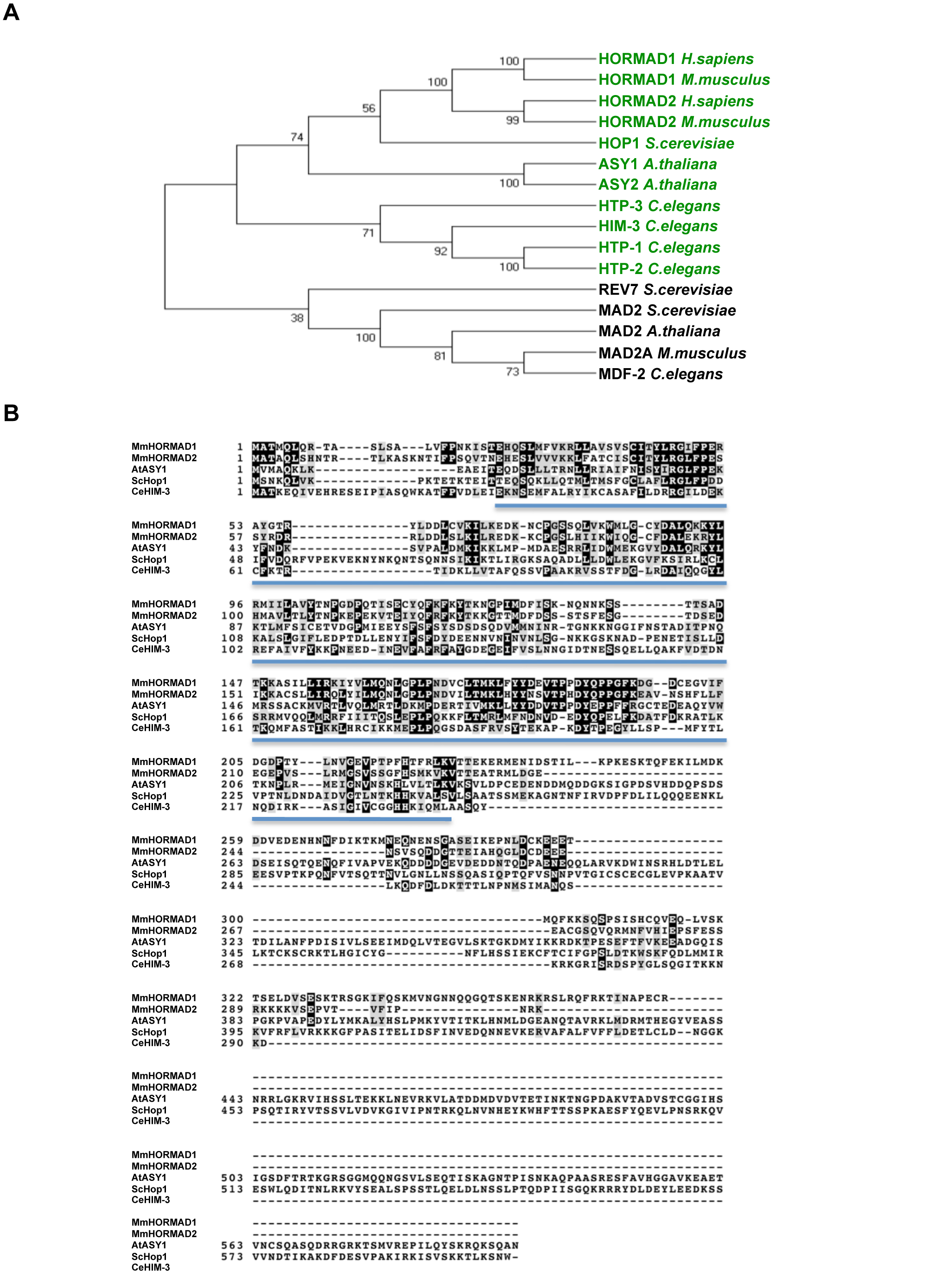

Supplement: Figure S2 — HORMAD1 and HORMAD2 are related to Hop1-like HORMA-domain proteins. (A) Phylogenetic tree of HORMA-domain containing proteins. The meiosis-specific Hop1 branch of HORMA-domain proteins is marked in green. Numbers are bootstrap values (see Materials and Methods). The full length amino acid sequences were used for the analysis. Accession number of each protein is presented in Table S1. (B) Alignment of Mus musculus HORMAD1 and HORMAD2, Arabidopsis thaliana ASY1, Saccharomyces cerevisiae Hop1 and Caenorhabditis elegans HIM-3 proteins. Black: Identical amino acids. Gray: Similar amino acids. The conserved HORMA-domain region is underlined [24]. (1.57 MB TIF) [file pgen.1000702.s002.tif]

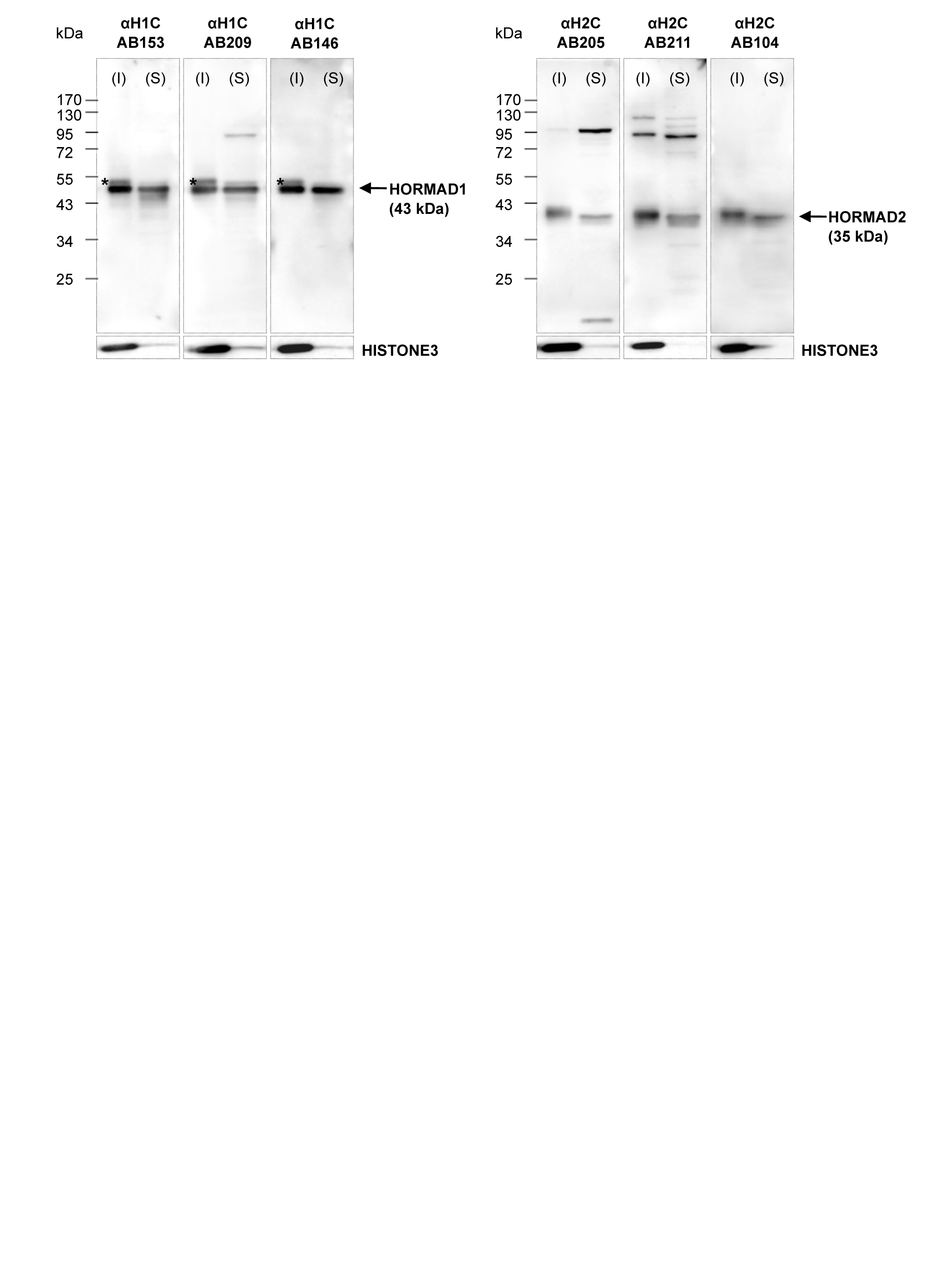

Supplement: Figure S3 — No cross-reactivity is observed between anti-HORMAD1 and anti HORMAD2 antibodies on immunoblots (IB). Detergent insoluble (I) and NP-40 soluble (S) fractions of 20 dpp mouse testis extracts were prepared as described in Materials and Methods. Following SDS-PAGE, immunoblot analysis was used to determine the molecular weight of proteins recognized by affinity-purified antibodies raised against the C-terminus of HORMAD1(αH1C) and HORMAD2 (αH2C). Fractionation of testis extracts was controlled by detection of HISTONE3 on all blot membranes. αH1C and αH2C antibodies recognize different proteins. All three αH1C antibodies (rabbit polyclonal AB209 and AB153 and guinea pig polyclonal AB146) recognized a protein which migrates slightly slower than what is predicted for HORMAD1. The additional, slower migrating protein detected by all of our αH1C antibodies in the detergent-insoluble fraction (*) is a phosphorylated form of HORMAD1 (our unpublished results). All αH2C antibodies (rabbit polyclonal AB205 and AB211 and guinea pig polyclonal AB104) recognized a protein which migrates slightly slower than what is predicted for HORMAD2. (0.43 MB TIF) [file pgen.1000702.s003.tif]

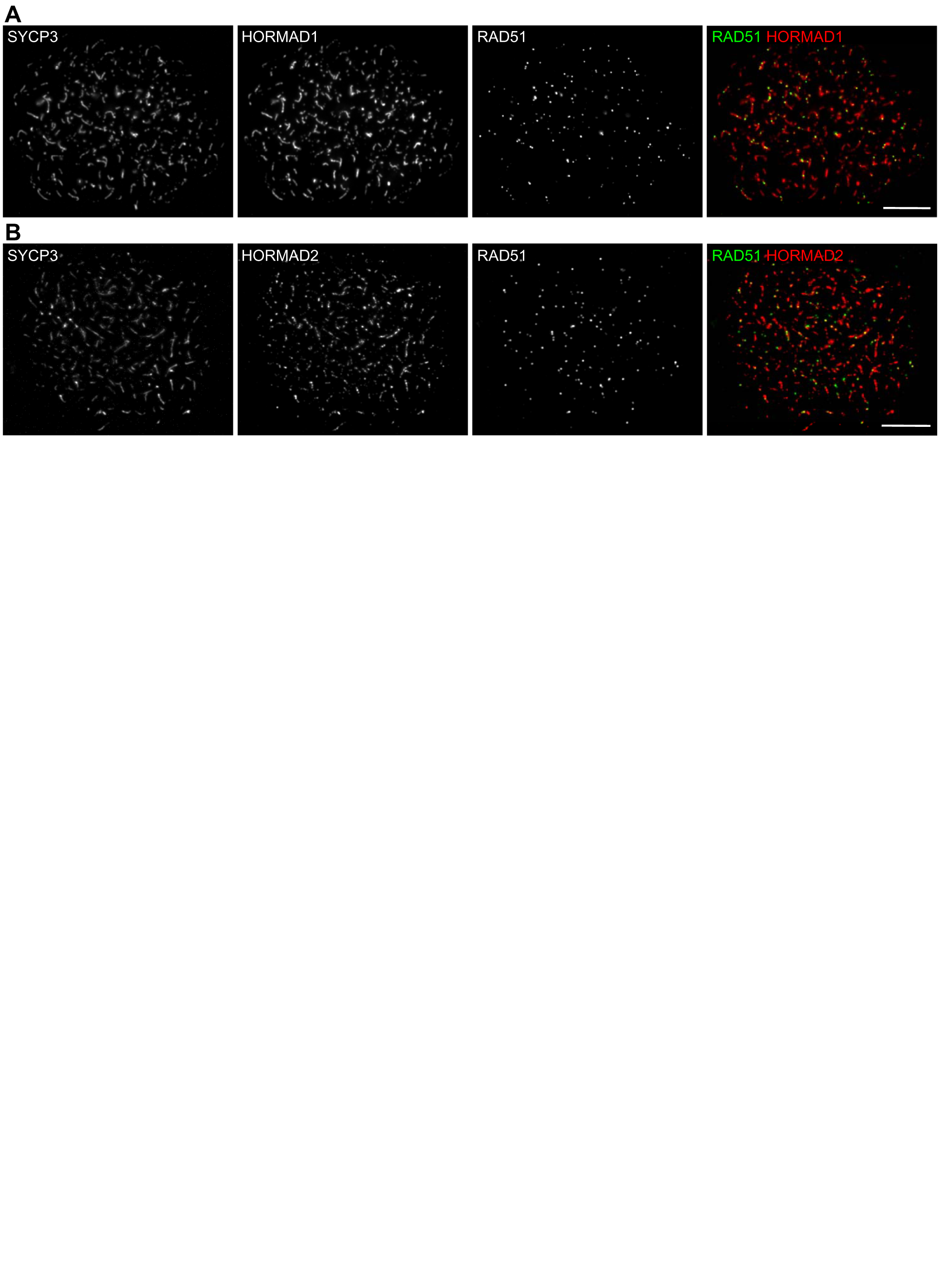

Supplement: Figure S4 — RAD51 foci closely associate with HORMAD1- and HORMAD2-associated axes during leptotene/early zygotene. SYCP3, RAD51, and either HORMAD1 (A) or HORMAD2 (B) were detected on nuclear spreads of leptotene/early zygotene spermatocytes. RAD51 foci are closely associated with forming axes decorated with HORMAD1 and HORMAD2. Bars, 10 µm. (1.30 MB TIF) [file pgen.1000702.s004.tif]

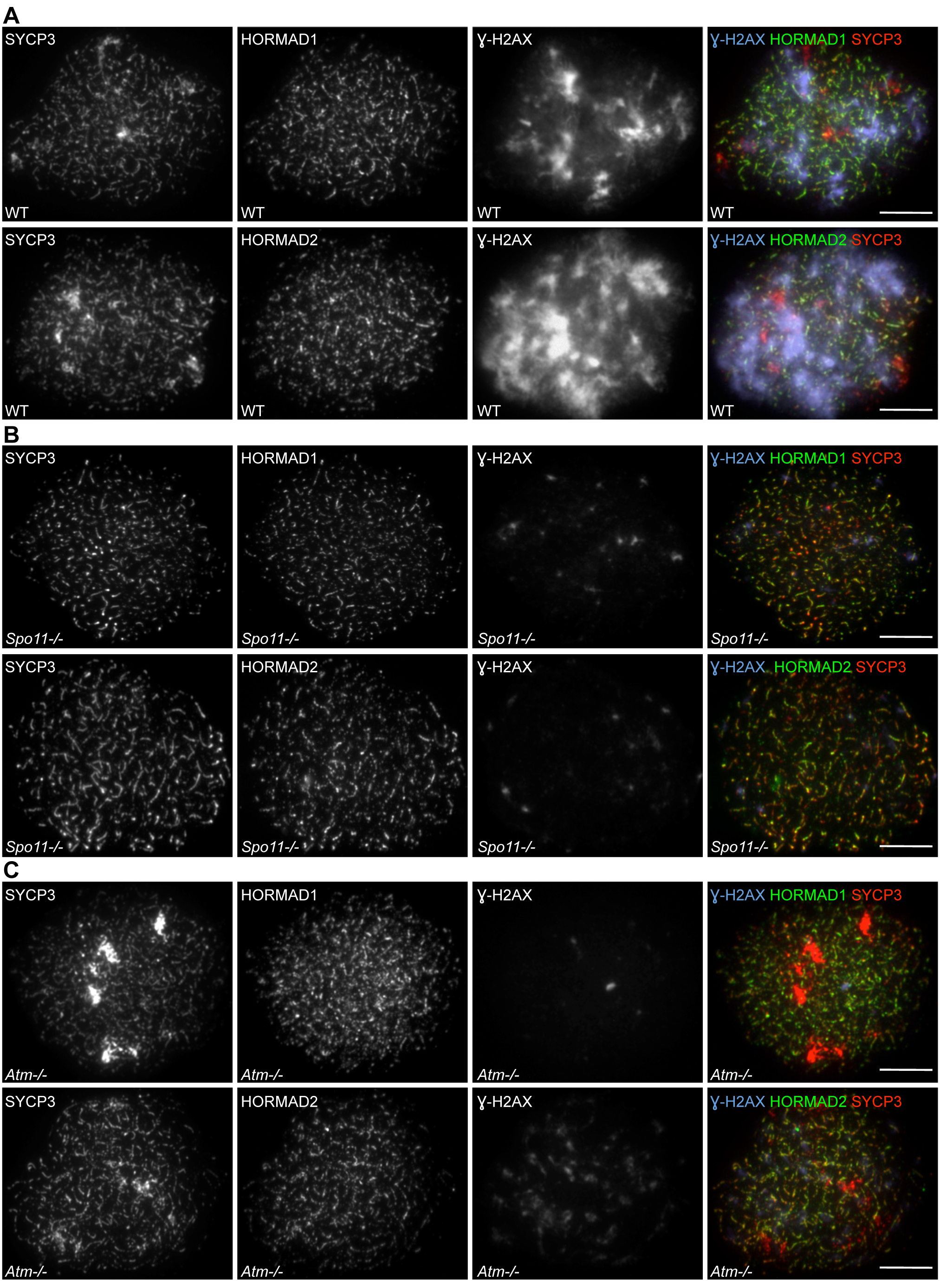

Supplement: Figure S5 — Localization of HORMAD1 and -2 to late leptotene and early zygotene chromosome axes is independent of DSB formation and ATM. Indicated proteins were detected by IF on nuclear surface spreads of WT (A), Spo11−/− (B) and Atm−/− (C) spermatocytes. Images were taken with the same camera settings to facilitate comparison of protein levels. Bars, 10 µm. (A) HORMAD1 and -2 appear on developing chromosome axes during leptotene in WT cells. In response to DSB formation ATM kinase promotes accumulation of γ-H2AX on chromatin at the time of axis formation. (B,C) HORMAD1 and -2 accumulate on the developing chromosome axes during leptotene in the absence of DSBs and ATM kinase activity (n = 100 cells). Accumulation of γ-H2AX on chromatin requires both DSBs and ATM during this early stage of prophase. (4.23 MB TIF) [file pgen.1000702.s005.tif]

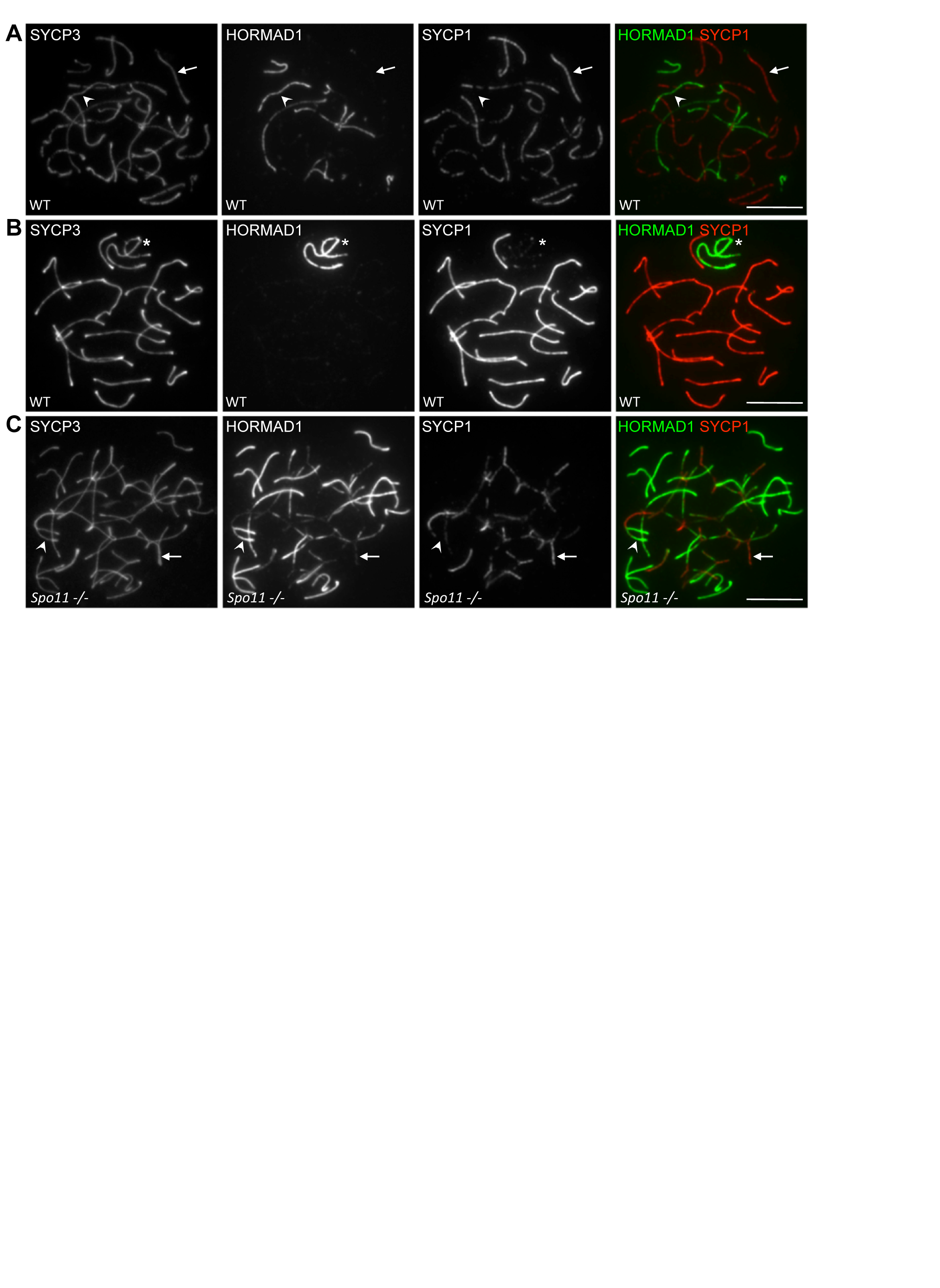

Supplement: Figure S6 — HORMAD1 levels on synapsed and unsynapsed axes are higher in Spo11−/− spermatocytes than in WT zygotene cells. SYCP3, SYCP1, and HORMAD1 were detected by IF on nuclear spreads that were prepared in parallel from WT (A and B) and Spo11−/− (C) testes. HORMAD1 and SYCP1 staining were compared on matched exposures of 30 randomly picked nuclei in two independent experiments. Representative images of WT zygotene (A), WT pachytene (B) and Spo11−/− (C) spermatocytes are shown. SYCP1 levels on non-homologously synapsed axes in the Spo11−/− mutant (C) are comparable to levels on homologously synapsed axes in WT zygotene cells (A) but are much lower than in WT pachytene cells. HORMAD1 signal on both unsynapsed and synapsed axes is higher in Spo11−/− cells than for the corresponding synaptic configurations in WT zygotene cells. Nevertheless, HORMAD1 signal in the mutant cells is substantially reduced on synapsed axes as compared to unsynapsed axes (n = 100 cells examined). The asterisk marks the sex chromosomes in the WT pachytene cell (A middle row). Examples are indicated of synapsed (arrows) and unsynapsed (arrowheads) axes. Bars, 10 µm. (1.37 MB TIF) [file pgen.1000702.s006.tif]

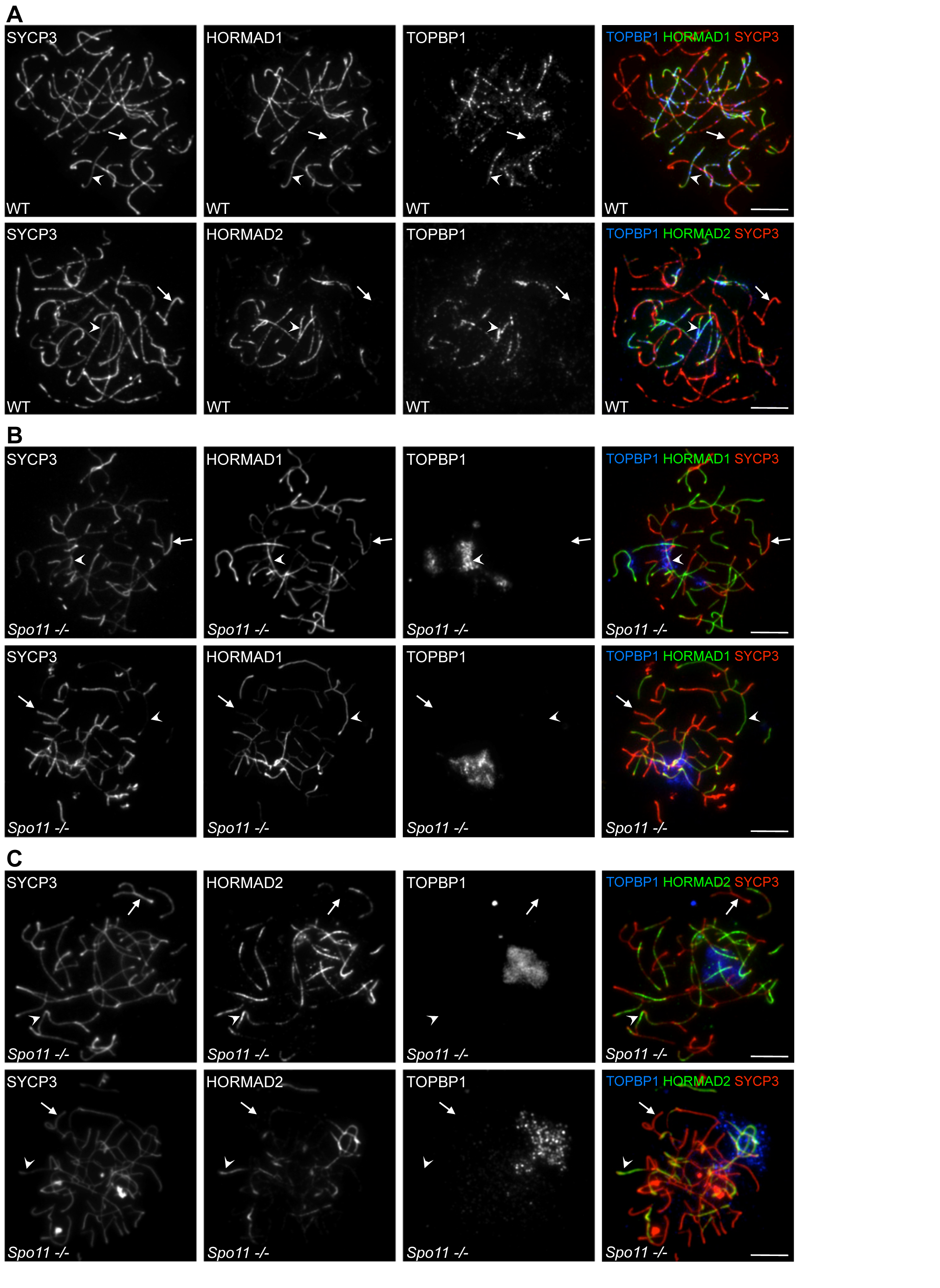

Supplement: Figure S7 — Localization of HORMAD1 and HORMAD2 in relation to TOPBP1. SYCP3, TOPBP1, and either HORMAD1 or HORMAD2 were detected by IF on nuclear spreads of spermatocytes from WT or Spo11−/− mutant testes. Bars, 10 µm. (A) Both HORMADs and TOPBP1 decorate unsynapsed axes in zygotene spermatocytes. Note that whereas HORMAD1 and -2 staining appears relatively continuous along unsynapsed chromosome axes, TOPBP1 instead forms dot-like foci. (B,C) In Spo11−/− spermatocytes, HORMADs preferentially localize to unsynapsed chromosome axes both within and outside of TOPBP1-rich regions, which correspond to pseudo-sex bodies [15]. In 9 out of 49 (18%) and 36 out of 55 (65%) “pachytene-like” cells (i.e., cells with locally restricted TOPBP1 accumulation (pseudo-sex body) and extensive synapsis), HORMAD1 and HORMAD2 hyper-accumulate in TOPBP1 rich regions, respectively. In the top rows of B and C, cells are shown in which HORMAD levels are comparable on unsynapsed axes inside and outside of TOPBP1-rich regions. In the bottom rows of B and C, cells are shown in which HORMADs hyper-accumulate within TOPBP1-rich regions. Examples are indicated of synapsed (arrows) and unsynapsed (arrowheads) axes. Bars, 10 µm. (2.44 MB TIF) [file pgen.1000702.s007.tif]

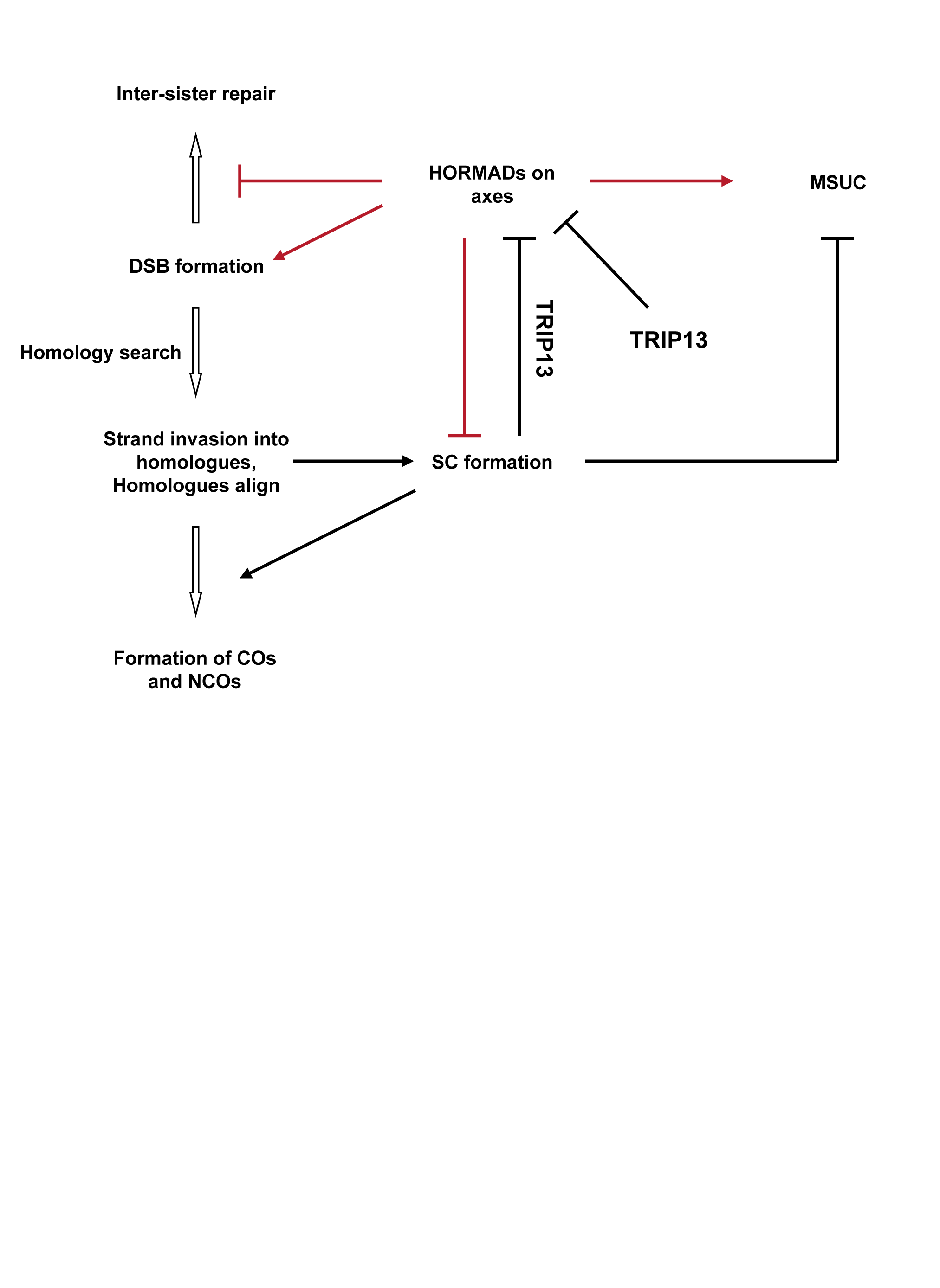

Supplement: Figure S8 — Speculative working model for the functions of HORMADs during male meiosis. Open block-arrows represent processes, flat-end red arrows represent inhibition (direct or indirect) and red arrows represent activation (direct or indirect). Possible functions of axis-associated HORMADs may include: promoting DSB formation, inhibiting inter-sister repair of DSBs, inhibiting promiscuous SC formation, and collaborating with ATR in promoting MSUC (see text for details). Inhibition of inter-sister repair permits the use of resected DSB ends for homology search. Stable strand invasion of DSB ends into homologous DNA sequences and chromosome alignment promotes legitimate SC formation. In turn, SC facilitates repair of DSBs as crossovers or noncrossovers in mammals [10]. SC also promotes depletion of HORMADs from chromosome axes in collaboration with TRIP13 (SC and TRIP13 could act independently or in the same pathway). Because homology searching is no longer necessary after SC formation, it is plausible that synapsis is accompanied by down-regulation of DSB formation and of inhibition of inter-sister DSB repair. HORMAD depletion from synapsed axes may help to accomplish this down-regulation. Depletion of HORMADs from synapsed autosomes may be one mechanism to restrict MSUC to unsynapsed sex chromosomes, thereby promoting progression past the mid pachytene checkpoint in males. Even if HORMADs promote MSUC, SC formation is able to inhibit ATR activity independently from the depletion of HORMADs from chromosome axes: despite abnormal persistence of low levels of γ-H2AX along synapsed autosomes in Trip13hypo pachytene cells, γ-H2AX preferentially accumulates on unsynapsed sex chromosomes [61]. (0.15 MB TIF) [file pgen.1000702.s008.tif]

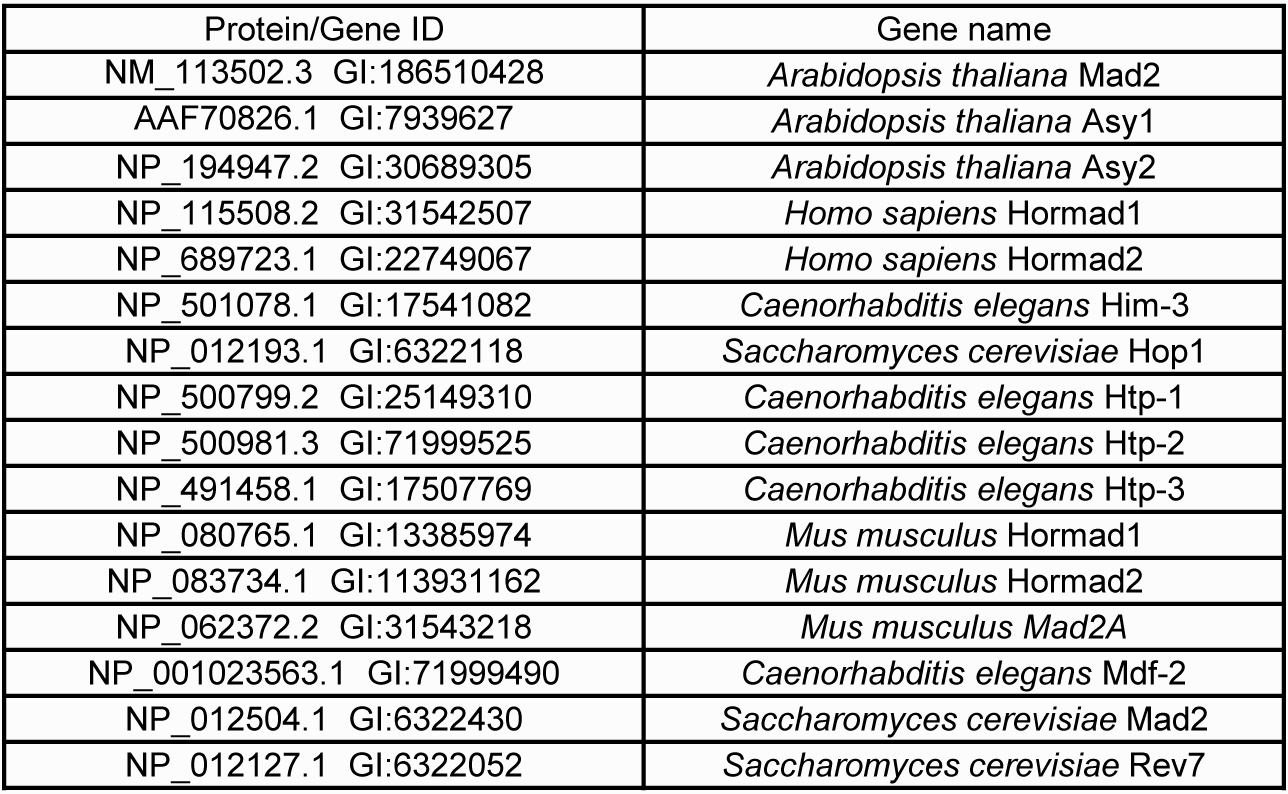

Supplement: Table S1 — Genbank accession numbers of HORMA-domain proteins used for phylogenetic comparison in Figure S2. (0.22 MB TIF) [file pgen.1000702.s009.tif]
